# Asymmetric membranes for gas separation: interfacial insights and manufacturing†

**DOI:** 10.1039/d3ra00995e

**Published:** 2023-05-10

**Authors:** Sharifah H. Alkandari, Jasmine Lightfoot, Bernardo Castro-Dominguez

**Affiliations:** a Centre for Advanced Separations Engineering, Department of Chemical Engineering, University of Bath Bath BA2 7AY UK bcd28@bath.ac.uk +44 (0)1225384946

## Abstract

State-of-the-art gas separation membrane technologies combine the properties of polymers and other materials, such as metal–organic frameworks to yield mixed matrix membranes (MMM). Although, these membranes display an enhanced gas separation performance, when compared to pure polymer membranes; major challenges remain in their structure including, surface defects, uneven filler dispersion and incompatibility of constituting materials. Therefore, to avoid these structural issues posed by today's membrane manufacturing methodologies, we employed electrohydrodynamic emission and solution casting as a hybrid membrane manufacturing method, to produce ZIF-67/cellulose acetate asymmetric membranes with improved gas permeability and selectivity for CO_2_/N_2_, CO_2_/CH_4_, and O_2_/N_2_. Rigorous molecular simulations were used to reveal the key ZIF-67/cellulose acetate interfacial phenomena (*e.g.*, higher density, chain rigidity, *etc.*) that must be considered when engineering optimum composite membranes. In particular, we demonstrated that the asymmetric configuration effectively leverages these interfacial features to generate membranes superior to MMM. These insights coupled with the proposed manufacturing technique can accelerate the deployment of membranes in sustainable processes such as carbon capture, hydrogen production, and natural gas upgrading.

## Abstract 2

Asymmetric membranes were manufactured *via* electrohydrodynamic emission, displaying enhanced performance compared to mixed matrix membranes. These membranes leveraged MOF–polymer crystalline interfaces to promote the separation of gas.

## Graphical abstract

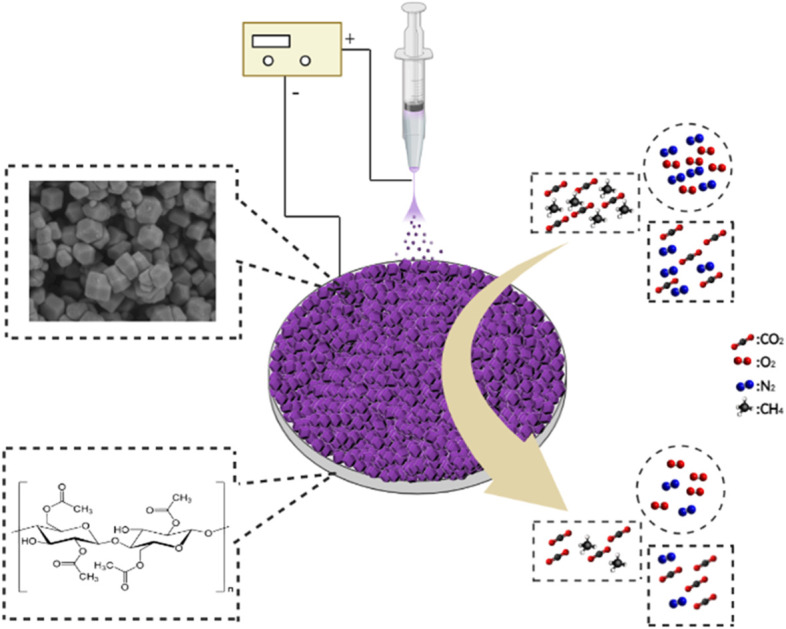

## 1 Introduction

The many advantages of membranes used for separation processes have led to its emergence as the preferred gas separation technique. Membranes are thin barriers that allow certain gas species to permeate through, while restricting others. These barriers display physicochemical properties that can be used for gas separation, including differences in gas solubility and diffusivity, a wide range of molecular pore sizes, and diverse gas adsorption affinities. Today, the most common materials exploited for gas separation are polymers, as they can be processed to attain large surface areas required for large scale processes. Unfortunately, polymers suffer from a trade-off between their gas selectivity and permeability.^1^ To circumvent this issue, membrane research has focused on developing novel composite materials (*e.g.*, asymmetric and mixed matrix membranes (MMM)) formed by a polymeric matrix containing disperse fillers that can help at sieving/adsorbing molecules or increasing the effective diffusivity of certain gases.^2,3^ Historically, most fillers have been micro-/nano-inorganic particles; however, the use of metal–organic frameworks (MOFs) has skyrocketed in the last two decades. MOFs are metal ions coordinated to organic ligands, which create frameworks with tunable pore sizes. Due to their properties, these highly porous structures are considered “A Material to Save the World”.^4^ According to the Web of Science, there are ∼7000 scientific papers related to polymers and membranes for gas separation, >40% of these are on MMM, and ∼1000 contain MOFs. Even so, as described by Beuscher *et al.*,^5^ “[…] research in membrane separation has focused on developing better membrane materials, yet very few of these materials are being used in commercial applications”.

There are various composite membrane manufacturing methods, namely (i) physical blending, (ii) sol–gel, and (iii) infiltration.^6–8^ For instance, the literature has shown many examples where MMM are fabricated *via* “physical blending”, where the polymers and MOFs are mixed and dispersed all together. Although, membranes manufactured in this manner have displayed reasonable separation performance; there are various challenges encountered, including filler agglomeration, filler size and interfacial morphology (*e.g.*, interface voids, sieves-in-a cage or a rigidified polymer layer around the fillers).^9,10^ Moreover, blending polymers with MOFs in solution remains an issue due to solvent compatibility and the possibility of the degradation of the MOFs supramolecular structure. The “sol–gel method” has displayed some advantages over physical blending;^7,11^ nevertheless, the range of applicable filler materials is comparatively narrow. For example, the center atoms of sol–gel precursors are limited to silicon and metal, nonetheless, precursors for carbon materials are rarely available. Additionally, the difficulty of synthesizing fillers *in situ* with multi-scale structures and multiple functionalities remains a challenge. These issues make this technique non-feasible for large scale membrane fabrication.^7^ Furthermore, the “infiltration method” consists of a strategy where the filler is synthesized after membrane formation. In this method, the precursor of the filler is permitted to infiltrate into a swollen or nano-porous polymeric membrane, thereafter the composite membrane is obtained by *in situ* filler growth and polymer curing.^12^ The limitation exhibited by this method is that the diffusion resistance along the narrow channels in the polymer matrix impedes the uniform distribution of the precursors. Hence, leading to a concentration gradient between the membrane surface and the inner center, which results to the enrichment of the fillers on the surface without any control during the process.^7,12^

To circumvent the challenges posed by today's membrane manufacturing methodologies, we propose to use electrohydrodynamic emission (EHE) as a technique to produce well dispersed, and stable MOF/polymer asymmetric membranes for gas separation. The EHE possesses a unique characteristic that enables controlling the dispersion, particle size distribution, supramolecular structure of crystals, surface topography, morphology, thickness, and other functional properties that determine membrane performance.^13^ EHE has the potential to deliver membranes at reduced costs, enhanced speed, quality, and consistency.^14^ In one study, Chowdhury *et al.* reported the capacity of EHE to control membrane thickness and smoothness using *M*-phenylenediamine (MPD) and 1,3,5-benzenetricarbonyl trichloride (TMC). The molar concentration of the MPD and TMC was held at a constant ratio of 4 : 1. They obtained composite membranes with thickness as low as 15 nm by electrospraying monomers (MPD and TMC) directly onto a substrate, where they reacted to form polyamide.^15^ Likewise, some other authors employed the EHE method for the fabrication of thin size composite membranes, thin films, and nanoparticles. However, most of the reported application of EHE for composite membrane fabrication was in the form of MMM, whereby the MOFs was mixed with the polymer solution and then electrospun.^16,17^

In this work, a new MOF printing methodology is proposed, leveraging EHE as a manufacturing technology, to fabricate asymmetric membranes. Asymmetric membranes are anisotropic structures consisting of a support and separation layer with distinct properties, such as permeability and morphology. The properties of either the support or selective layer can be modified or optimized independently without significantly increasing the overall membrane cost.^18,19^ Asymmetric membranes are operated with a selective layer exhibiting sufficient mechanical strength, dense surface and largest pores facing upstream. Here, we studied ZIF-67/CA membrane system *via* molecular simulations to demonstrate the advantageous features of the asymmetric configuration and simultaneously elucidating – at a molecular level – their gas separation mechanism.

## 2 Methodology

This work shows the development of a cellulose acetate (CA)–zeolitic imidazolate framework 67 (ZIF-67) membrane for the separation of CO_2_ from natural gas, and N_2_ from air. CA was chosen due to its commercial membrane fabrication relevance^20^ and its excellent CO_2_ solubility.^21^ ZIF-67 was chosen because it has high affinity for CO_2_, exhibits large surface area and porosity,^22^ adjustable pore sizes and possibilities for surface property functionalization. More so, it is characterized with a flexible framework, low densities (0.2–1 g cm^−3^), and significant thermal/chemical stability^23^ in addition to the presence of organic ligands in their structure which enhances affinity and adhesion with polymers and other organic materials.^24^ Furthermore, ZIF-67 possesses a pore size of 0.34 nm, which falls between the kinetic diameter (*d*_k_) of CO_2_ (0.33 nm) and larger molecules such as N_2_ (0.364 nm) and CH_4_ (0.38 nm).^25,26^ The experimental conditions and parameters employed in this study were chosen based on trial-and-error experiments, as well as recommendations derived from the literature.

### 2.1 Materials

Acetone, *N*,*N*-dimethylacetamide (DMAc) and cellulose acetate (CA) with 39.85% acetyl content and number-average molecular weight of 3.0 × 10^4^ Da were supplied by Aldrich Chemical Co. Inc. in a fine, dry, and free-flowing powder form. Cobalt nitrate hexahydrate (Co(NO_3_)_2_·6H_2_O, 99% purity), 2-methylimidazole (MeIm, 99% purity), methanol (CH_3_OH, analytical reagent), and deionized water were purchased from Merck. All materials were used without further purification or treatment.

### 2.2 CA membrane fabrication

The solution casting method was used for the fabrication of the CA membrane. The preparation of the dope CA solution, which included solvent mixture ratio, temperature, stirring time, and wait-on time; the parameters were drawn from several studies, including Febriasari *et al.* 2021, Omollo *et al.* 2014, Ahmad *et al.* 2014, and Liu *et al.* 2002.^27–30^ The CA dope solution was prepared by dissolving 15 wt% in a solvent containing acetone and DMAc in a 2 : 1 ratio. The solution was homogenized in a round sealed glass container by stirring for 24 h, until a clear solution was observed. The solution was kept for 12 h to remove all possible air bubbles. The CA solution was cast and kept for 24 h under atmospheric temperature before placing the formed layer in a heated oven at 130 °C overnight, as shown in Fig. S1.† To determine the critical concentration of CA reported in this study, various concentrations of CA (ranging from 5 to 25 wt%) were fabricated, characterized, and subjected to gas permeation studies for optimization.

### 2.3 ZIF-67 synthesis

ZIF-67 crystals were fabricated following the procedure reported by Feng *et al.* 2020.^31^ Cobalt nitrate hexahydrate (Co(NO_3_)_2_)·6H_2_O (1.0 g) and polyvinylpyrrolidone PVP (0.85 g) were dissolved in a solution containing 60 mL methanol to form solution A. Afterwards, 4.0 g of 2-methylimidazole (MeIm; C_4_H_6_N_2_) was dissolved in another solution containing 60 mL of methanol to form solution B. Then solution B was poured into solution A under continuous stirring for 10 minutes for homogenization and aged for 24 h at room temperature. The resultant precipitates were centrifuged for collection and washed with methanol six times. Finally, the ZIF-67 particles with size of 340 nm were vacuum-dried at 100 °C for 24 h.

### 2.4 ZIF-67/CA membrane fabrication

**Fig. 1:**
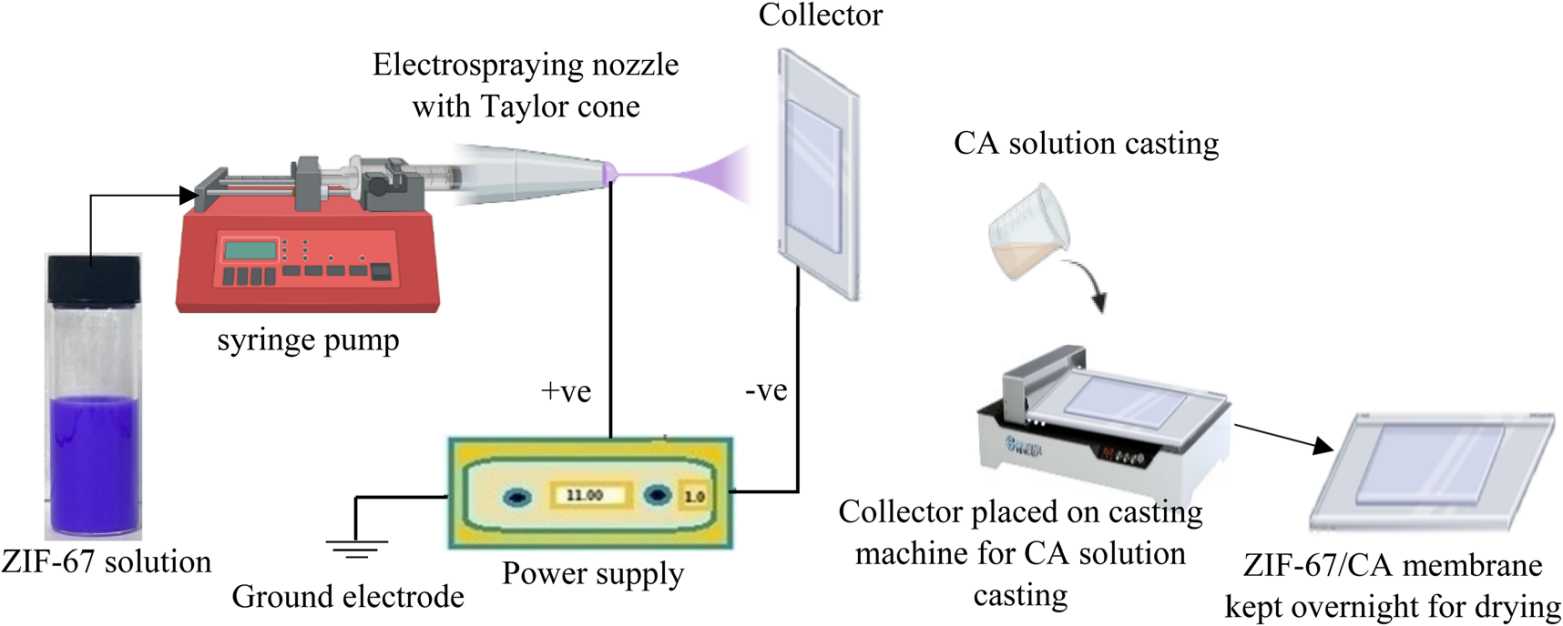
Schematic procedure of ZIF-67/CA asymmetric membrane fabrication.

The ZIF-67 solution was prepared by dispersing 50 mg of the synthesized ZIF-67 particles in 6 g of DMAc and homogenized by continuously stirring for several hours to produce a ZIF-67 suspension. CA equivalent to 1 wt% of the ZIF-67 particles was added to the ZIF-67 suspension and homogenized. CA was used as glue for the ZIF-67 particles, thereby improving the ZIF-67/CA compatibility. The ZIF-67 suspension was then ultrasonicated for 30 min in a water bath to ensure proper dispersion. Subsequently, the suspension was loaded into a syringe with an internal diameter of 5.19 mm, mounted on a precision syringe pump that was used to regulate the feed rate during electrospraying (see Fig. 1). The grounded electrode from high voltage power supply is intended to generate a potential difference between the nozzle tip and the collector. The Taylor cone nozzle tip was positioned at 8 cm from the collector, and the ZIF-67 suspension was electrosprayed at a constant flowrate of 0.015 mL min^−1^ and a voltage of 10–12 kV. Once the electrosprayed ZIF-67 is uniformly distributed on the collector, CA solution was casted on top of the ultrathin ZIF film. The asymmetric membrane was left in the collector at controlled humidity and temperature for 24 h to allow evaporation. Thereafter, the asymmetric ZIF-67/CA membrane was peeled off from the collector to obtain a typical free-standing membrane. Finally, the membrane was placed in a vacuum oven at 130 °C overnight to completely evaporate any remaining solvent. For comparison, mixed matrix membranes (MMM) were fabricated by adding 4.10 wt% of ZIF-67 into a solution which is made up of acetone and DMAc in a 2 : 1 ratio. The solution was then mixed and sonicated for 3 h. Thereafter, 15 wt% of CA was added in three different batches, with each addition followed mixing, then sonication. Afterwards, the solution was kept overnight for homogenization, and subsequently sonicated. For deaeration, the ZIF-67/CA solution was kept for 12 h prior to casting. After casting, the cast solution was left under atmospheric temperature for 24 h. Thereafter, the fabricated ZIF-67/CA MMM was placed in a heat oven and heated overnight at 130 °C.

### 2.5 Characterization

#### 2.5.1 X-ray diffraction (XRD)

The crystalline properties of the synthesized ZIF-67 MOF, the pristine CA polymeric membrane and the ZIF-67/CA asymmetric membrane, were measured using powder X-ray diffraction. The crystallinity and solid phase structure of the samples were recorded at room temperature on a Bruker D2 PHASER diffractometer operated at 40 kV and 40 mA using CuKα radiation with wavelength (*λ*) = 1.54059 Å. XRD scans were made from 5° to 50° 2-theta with a step size of 0.02° and a scan speed of 0.2 second per step.

#### 2.5.2 Thermogravimetric analysis

Thermal gravimetric analysis (TGA) measurements were performed to determine the thermal stability of the synthesized ZIF-67 MOF and CA polymer using a Setaram Setsys Evolution 16 TGA. Prior to the analysis, the samples were dehydrated and degassed for 24 h under vacuum at 80 °C. Then, approximately 10 mg sample of each were introduced into an alumina crucible and heated under argon atmosphere from 20 °C to 800 °C at a ramp-up rate of 10 °C min^−1^.

#### 2.5.3 FT-IR analysis

Fourier Transform Infrared Spectroscopy (FTIR, Spectrum 100™ PerkinElmer USA), packaged with total reflectance cell ranging from 4000 cm^−1^ to 650 cm^−1^ was used to analyze the chemical structure and obtain the functional group details of the pristine CA membrane, the as-synthesized ZIF-67 and the fabricated ZIF-67/CA asymmetric membrane. Prior to testing the samples, a background scan was run in transmission mode at a spectra resolution of 4 cm^−1^ and the spectra was recorded for the total reflectance cell range.

#### 2.5.4 SEM-EDX analysis

The morphology of the synthesized ZIF-67, CA polymer and fabricated ZIF-67/CA asymmetric membrane were determined using a variable pressure Scanning Electron Microscopy SEM (SU3900, Hitachi, Japan), incorporated with energy-dispersive X-ray spectroscopy (EDX). The respective sample morphology was analysed by capturing the surface, as well as the cross-sectional SEM images.

#### 2.5.5 Gas sorption analysis

The gas sorption of the ZIF-67 nanoparticles was analyzed by examining its surface area and pore size distributions with nitrogen adsorption and desorption at 77 K using a Micromeritics 3Flex 3500, USA volumetric gas sorption analysis system. The Brunauer–Emmett–Teller (BET) method was applied for specific surface area calculation using regression analysis based on relative pressure data from 0 to 1.0, following the manufacturer's guidelines.

#### 2.5.6 Gas permeation tests

The permeation measurement of the fabricated membrane was done using the constant-volume/variable-pressure technique with a time-lag apparatus. The permeability of the single gases (CO_2_, CH_4_, N_2_ and O_2_) was measured at 25 °C, at feed pressure of 1 bar gauge. The schematic diagram of the experimental setup is shown in Fig. S2.† The measurement was repeated multiple times to validate the reproducibility of the results, and the final data was recorded after a steady state was attained. The gas permeability coefficient (*P* in barrer) was calculated from the slope along the steady-state regions by applying eqn (1). The ideal selectivity for binary gas pairs of A and B was determined from eqn (2).1
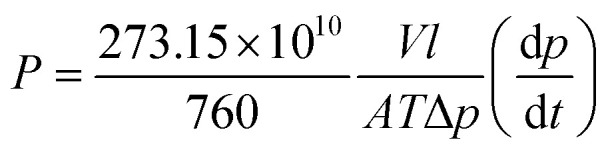
2
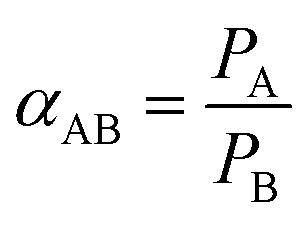
where *P* is the permeability represented in barrer (1 barrer = 10^−10^ [cm^3^(STP) cm]/(cm^2^ s cm_Hg_)); *V* (cm^3^) is the permeate volume; *l* (cm) is the membrane thickness; *A* (cm^2^) is the effective membrane area; *T* (K) is the operating temperature (K); Δ*p* (cm_Hg_) the pressure difference between the injection and the permeate sides; d*p*/d*t* is the steady state rate of pressure rise. *P*_A_ and *P*_B_ are the permeability coefficient of gases A and B, respectively. The more permeable gas is taken by default as the gas A, as such, *α*_AB_ > 1.

### 2.6 Molecular simulations

Structures of ZIF-67 were generated from crystallographic data, derived from X-ray diffraction. Bulk and slab systems of ZIF were built accordingly, using METADISE.^32^ Recent studies to determine force field terms of ZIF-67 were used in this model,^33^ and the TraPPE force field^34^ was implemented to model penetrating gas molecules. In generating the composite ZIF-67/CA system, 37 polymer chains were packed into a lengthened simulation cell containing a ZIF-67 slab. The generation and equilibration of polymer, ZIF and composite systems are detailed in the ESI.† The open-source molecular dynamics (MD) package, GROMACS was used to perform MD simulations.^35–37^ Free volume calculations were performed by extracting the configurational output of MD simulations and using the SCAN function of DL_MONTE,^38^ to perform systematic grand canonical Monte Carlo insertions of a hydrogen probe molecule at regular intervals throughout the simulation cell. The calculation of density, dihedral distribution, free volume, radius of gyration, radial distribution function, radii of gyration of CA molecules are described in detail in the ESI.†

## 3 Results and discussion

### 3.1 Structural and supramolecular analysis

**Fig. 2:**
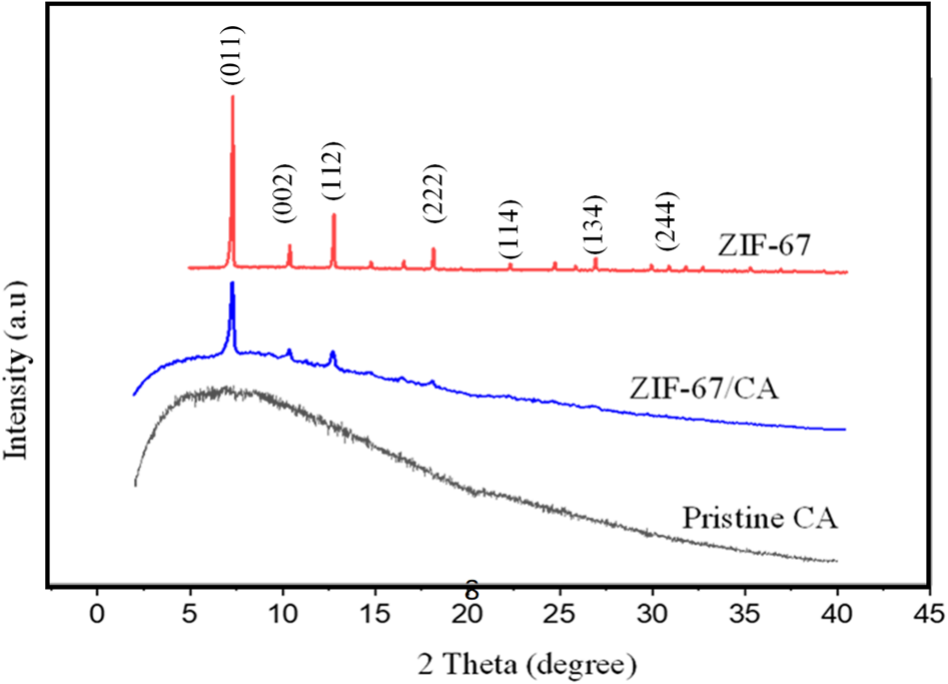
XRD analysis of pristine ZIF-67 particles, CA polymer and fabricated membrane with a 4.10 wt% loading.

Fig. 2 shows the powder X-ray diffraction pattern of the synthesized ZIF-67 particles, pristine CA and 4.10 wt% ZIF-67/CA asymmetric membrane. The XRD spectra of the ZIF-67 produced in this work resembles those reported in the literature,^39^ suggesting that the supramolecular structure of the ZIF-67 was successfully synthesized. The main peaks, assigned to the crystal faces, were observed at 7.28°, 10.38°, 12.66°, 17.87° and 32.87°. The intensity of the peaks at 7.32° and 10.38° indicates that the growth of ZIF-67 particle crystal-faces is high.^31,40^ The free volume of the synthesized ZIF-67 MOF was analyzed using low-pressure gas adsorption (N_2_) at a temperature of −195.615 °C with BET surface area of 1311 m^2^ g^−1^ (Fig. S3†).

The XRD pattern of the pristine CA shows an amorphous curve as indicated by the broad diffraction peaks in Fig. 2. This curve is characteristic to non-crystalline natural polymers. The CA spectra shows no prominent crystalline reflection peaks, which verifies its characteristic amorphous structure and agrees with the characteristic peaks of pristine CA previously reported in the literature.^21,41,42^ The asymmetric membrane of ZIF-67/CA shows an XRD curve that mirrors the characteristic peaks of ZIF-67 at 2-theta values of 7.28°, 10.38°, 12.66°, and 17.87° and the amorphous nature of the CA. This result suggests that the supramolecular structure of ZIF-67 remains stable after: (i) being exposed to the CA precursor solution; and (ii) the electrospray does not produce any structural changes to the particles. The polymer–MOF superimposed XRD spectra also suggests a good interaction between the CA polymer matrix and the ZIF-67 particles.

### 3.2 Thermal stability analysis

The thermal stability of synthesized ZIF-67, pristine CA and the asymmetric membrane of 4.10 wt% ZIF-67/CA membrane were thermogravimetrically analyzed in argon atmosphere. The TGA of pure ZIF-67 particles indicated good thermal stability, with a plateau and no significant weight loss recorded up to temperature values of 500 °C. However, at about 560 °C an obvious weight loss was observed, which is indicative of the thermal decomposition of the synthesized ZIF-67 nanoparticle. These results suggest that the thermal behavior of the produced nanoparticles corresponds to those of ZIF-67.^43–45^ Overall, the TG analysis indicates that the synthesized ZIF-67 has excellent thermal stability up to 560 °C.

**Fig. 3:**
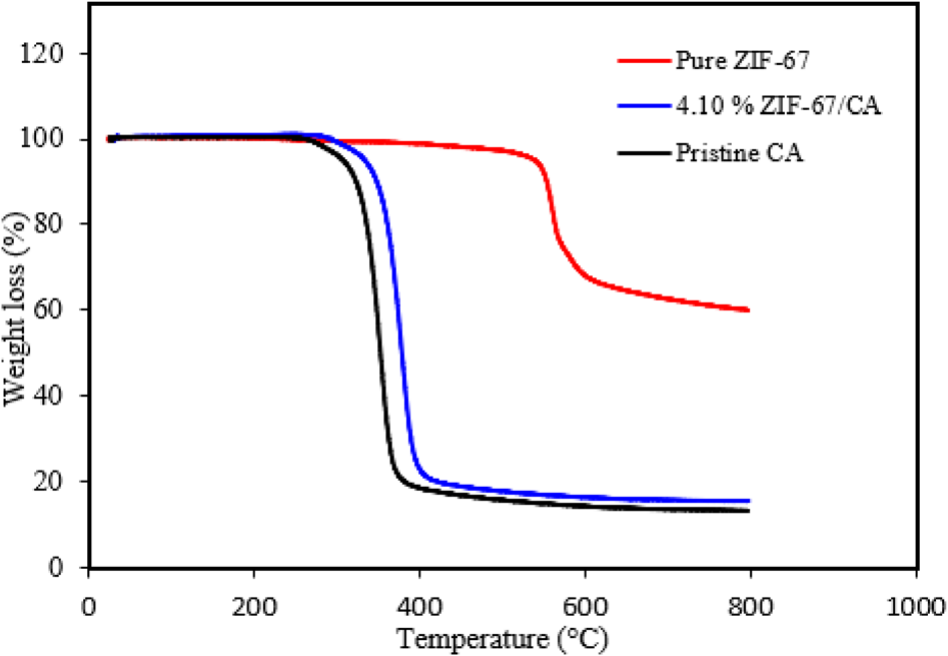
TGA result of synthesized pristine CA, pure ZIF-67 and 4.10 wt% ZIF-67/CA.

Similarly, the TG analysis curve of the pristine CA membrane and the asymmetric ZIF-67/CA membrane are presented in Fig. 3. As shown in the figure, both the pristine CA and the asymmetric ZIF-67/CA membrane have similar shape of the weight loss curve. However, some of the observable differences is that the combination of 4.10 wt% of ZIF-67 with the pure CA caused an increase in the thermal stability of the pure CA. For the pristine CA, the plateau is sustained until about 280 °C, whereas the asymmetric ZIF-67/CA membrane is at plateau till 293 °C. Likewise, beyond 400 °C, with the increase in the temperature, the remaining weight of the asymmetric membrane is higher than the pristine CA. This phenomenon is attributed to the excellent thermal stability of ZIF-67 that was added to the asymmetric membrane.

### 3.3 Chemical analysis of the pristine CA, ZIF-67, and asymmetric membrane

**Fig. 4:**
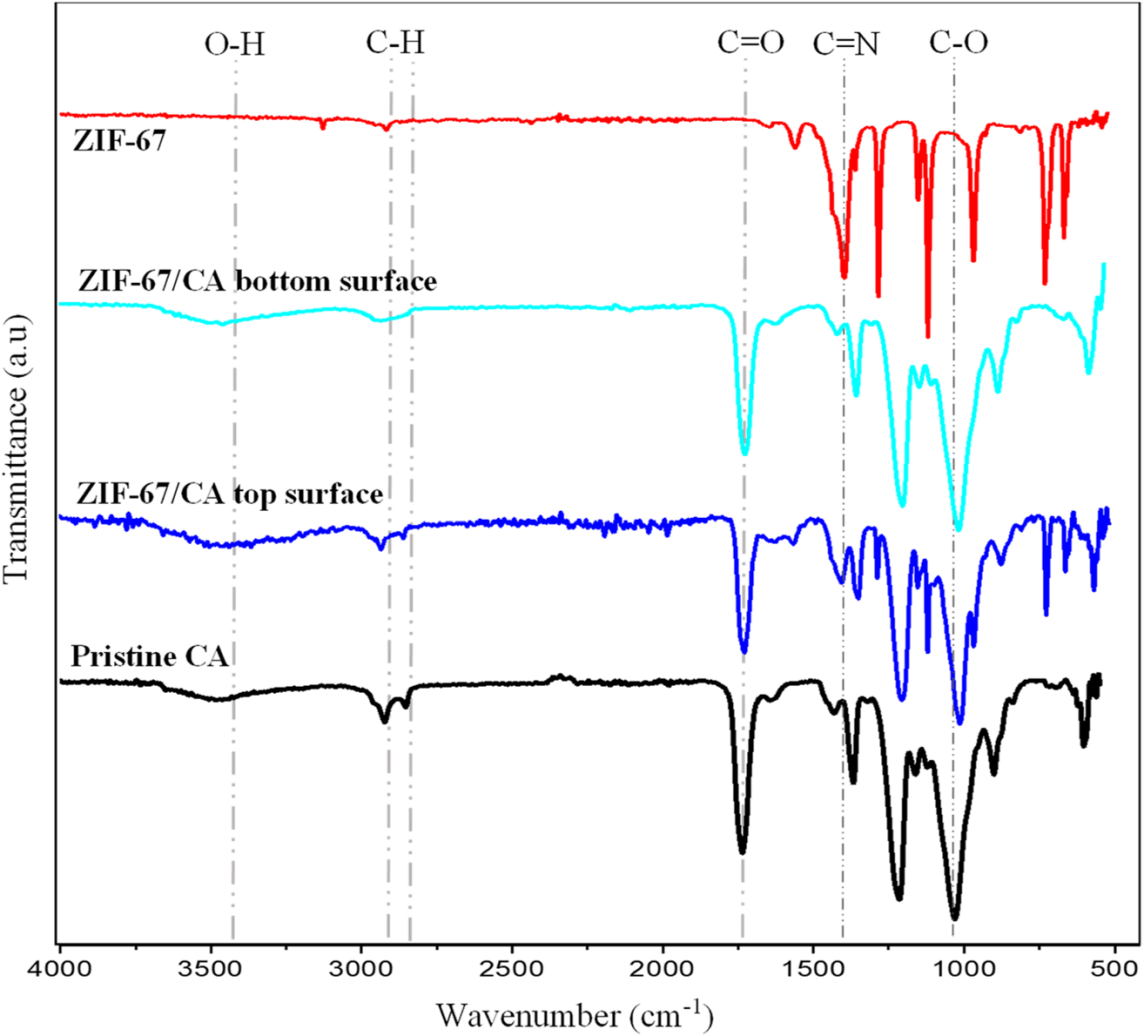
FT-IR spectra of pristine CA, ZIF-67 and 4.10 wt% ZIF-67/CA asymmetric membranes.

Fourier transform infrared spectroscopy (FTIR) spectra of the pristine CA, synthesized ZIF-67 particles and 4.10 wt%-ZIF-67/CA asymmetric membrane was obtained to confirm the presence of active functional groups. Fig. 4 shows the FTIR spectra of the synthesized ZIF-67, revealing absorption bands, attributed to the 2-methylimidazole ligand, and exhibited typical vibration absorbance bands identically positioned as those reported in the literature.^46–48^ The absorption bands ranging from 600 cm^−1^ to 1500 cm^−1^ show the stretching and bending of the imidazole group. Moreover, the absorption bands within the range of 1380 cm^−1^ to 1450 cm^−1^ are attributed to the stretching vibration of the whole imidazole group, while those ranging from 800 cm^−1^ to 1380 cm^−1^ are attributed to the in-plane bending vibration in the ring and those below 800 cm^−1^ correspond to the out-of-plane bending, respectively. The absorption bands observed at 1417 cm^−1^ are a resultant of the stretching condition of the C=N bonds in the 2-methylimidazole. More so, the stretching vibration of the alkane C–H from the aliphatic methyl group and aromatic ring of the 2-methylimidazole are the precursor of the peaks observed at 2926 cm^−1^ and 3135 cm^−1^, respectively. Similarly, the FTIR analysis of the pristine CA and both surfaces of the asymmetric ZIF-67/CA membrane depict spectrum identical to those reported by previous authors.^49,50^Fig. 4 shows that the stretching vibrations of alkane C–H bonds are observed within wavenumber of 2922.69 cm^−1^ and 2853 cm^−1^. Likewise, the peaks at 1735 cm^−1^ correspond to the carbonyl, C=O functional group vibration in the acetate substituent while those observed at wavenumber of 3485 cm^−1^ corresponds to the hydrogen oxygen, O–H vibration in hydroxyls or water present. The characteristic absorption bands observed in the pristine CA were similar to conventional spectra, which is an indication of no detectable chemical interaction with any other particle in the system. Likewise, the FTIR spectra of the bottom surface of the asymmetric membrane is a typical replica of the pristine CA, which further proves the absence of MOF particles at the bottom end, because the electrospray was on the top end. Whereas the consistency of the top surface of the asymmetric ZIF-67/CA membrane FTIR spectra with the pristine CA spectra indicated a good compatibility between the cast CA-based polymeric membrane and the electrosprayed ZIF-67 MOF.

### 3.4 Structure of the ZIF-67/CA asymmetric membranes

**Fig. 5:**
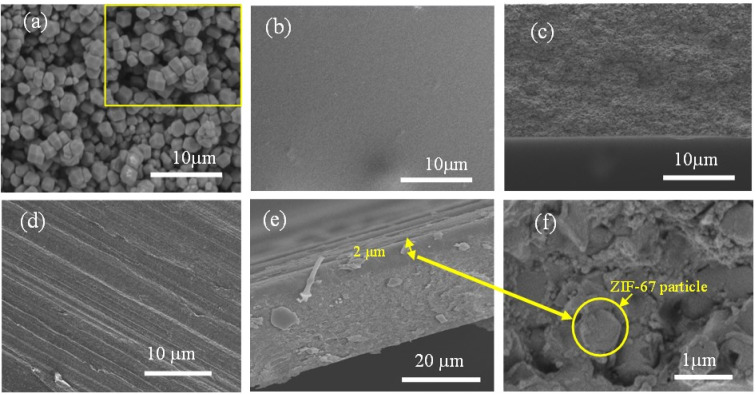
SEM images of: (a) ZIF-67 particles; (b) pristine CA surface; (c) pristine CA cross-section; (d) 4.10 wt% ZIF-67/CA surface; (e) 4.10 wt% ZIF-67/CA cross-section; and (f) 4.10 wt% magnified cross-section of ZIF-67/CA top layer.

The surface morphology of membrane can significantly impact the gas transport properties. Consequently, each membrane was subjected to SEM analysis. Fig. 5 shows SEM images depicting the morphology and geometry of the pristine CA, ZIF-67 particles, and the asymmetric 4.10 wt% ZIF-67/CA membrane. Fig. 5a shows that ZIF-67 is formed of monodispersed polyhedron particles with an average particle size of 340 nm. The crystals have no noticeable orientation, a typical characteristic of this MOF.^31,46^ The lack of orientation is further confirmed by the XRD patterns shown in Fig. 2. The surface and cross-sectional SEM image of the pristine CA membrane is presented in Fig. 5b and c, respectively. The pristine CA membrane showed a dense, smooth, and flat surface morphology, a characteristic feature found across the literature.^21,51^Fig. 5d shows the surface morphology of the ZIF-67/CA asymmetric membrane, while Fig. 5e and f show the cross-section and the magnified cross-section of the top layer, respectively. The cross-sectional images reveal that the membranes have an asymmetric structure, containing a smooth, thick CA-rich layer; and on top a thin layer of ZIF-67 nanoparticles with a thickness of 2 μm (see Fig. 5e and f, yellow double-head arrow and a yellow circle). The top layer is a thin asymmetric film containing ZIF-67 particles/CA with a significant concentration of MOF. It is important to note that the literature may have reported MMMs with total filler loading ≥4.10 wt% concentration, however, because the filler is dispersed and homogenized in the polymeric solution, the concentration of the filler on the separation surface is a fraction of the total amount dispersed. Therefore, this work has the highest reported pure filler concentration on the separation surface in the literature. Moreover, the SEM images show that the ZIF-67 particles are homogenously distributed, a result of the electrospray aerosolization mechanism. Even at high loadings, this electrosprayed layer did not change the topology of the membrane, nor displayed any form of surface defect or voids, like those typically observed and reported at high filler loadings.^52^

**Fig. 6:**
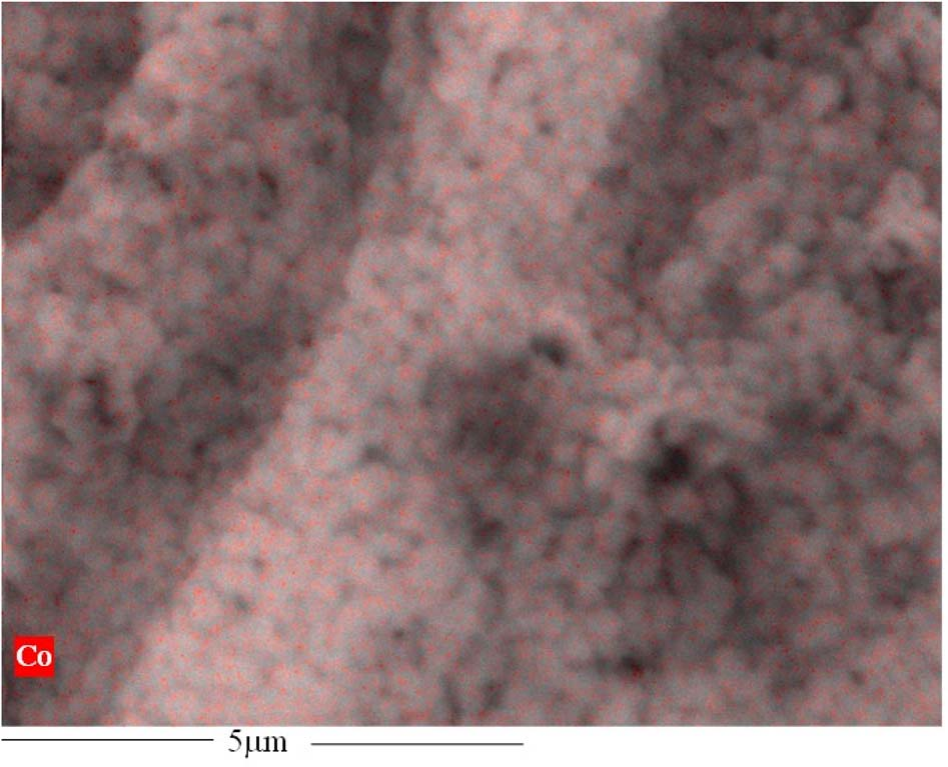
The EDX analysis of 4.10 wt% ZIF-67/CA membrane surface.

The ZIF-67 particle distribution was confirmed by energy-dispersive X-ray spectroscopy (SEM-EDX) analysis, as shown in Fig. 6 and S4a.† The analysis shows that the cobalt (Co) metal in ZIF-67 was homogenously distributed throughout the membrane surface, with no noticeable defects or large-scale phase variation. The absence of noticeable phase variation on the membrane surface is an indication of a good interface between the CA polymer and ZIF-67, which is caused by the presence of multifunctional organic ligands in the ZIF-67 structure. Similarly, the EDX mapping of the bottom surface of the asymmetric ZIF-67/CA membrane shows a smooth surface with no indication of defects, agglomeration or the presence of cobalt. This further verifies that the bottom surface is rich in CA, see Fig. S4b.† On the contrary, the MMM EDX mapping showed an obvious agglomeration in cobalt (Co) at different positions on the surface (Fig. S5a†).

**Fig. 7:**
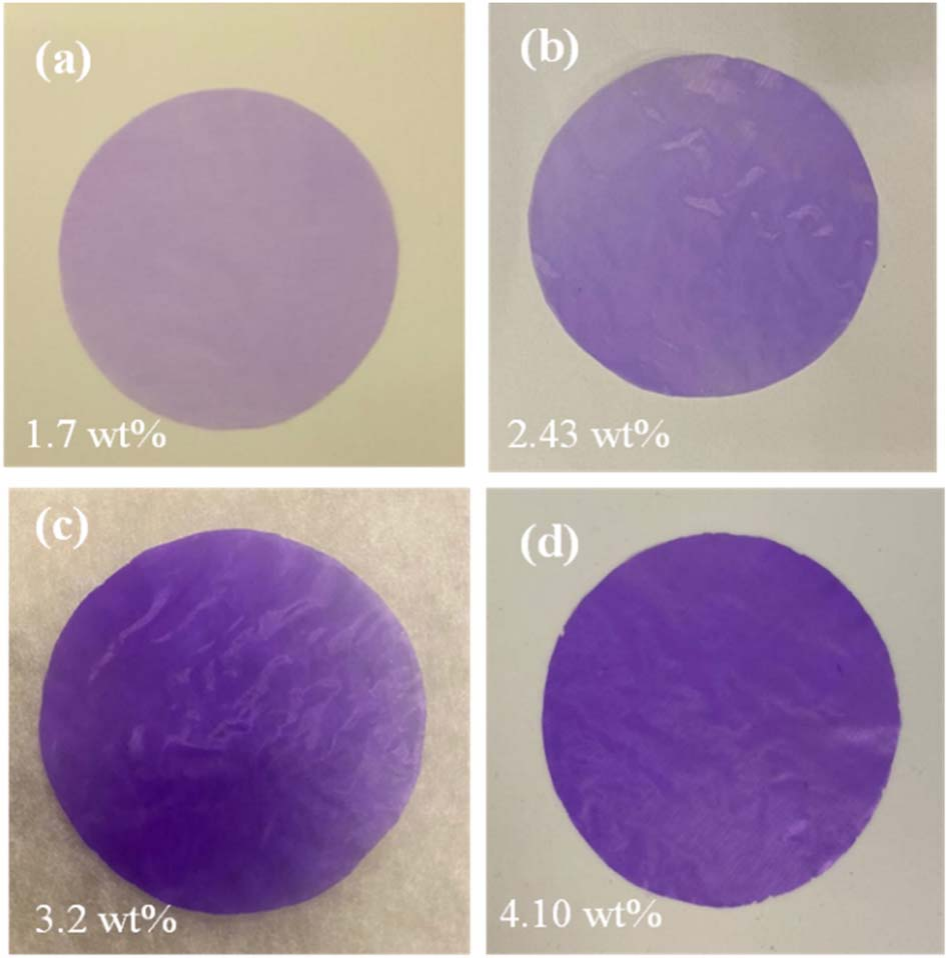
Optical photos of ZIF-67/CA membranes with ZIF-67 loading of (a) 1.7 wt%, (b) 2.43 wt%, (c) 3.2 wt%, and (d) 4.10 wt%.

Fig. 7a–d shows the homogeneity of the filler's dispersion, indicated by the evenly distributed pink/purple color of the MOF particles. The uniform distribution and absence of agglomeration of ZIF-67 particles at high concentrations suggest that EHE is an effective membrane manufacturing technique. During the EHE process, the high voltage applied to the MOF precursor generates an aerosol of small, highly charged droplets dispersed by Coulomb repulsion. This droplet surface charge effectively prevents agglomeration after spraying the precursor. The solvent of the droplets evaporates to yield monodispersed particles at the surface of the EHE collector. The thickness of the particles deposited on the membrane can be controlled by the concentration of the MOF in the precursor solution.

The authors suggest that future work should focus on: (i) exploiting the high concentration of MOF particles contained at the surface to increase their circularity and sustainable profile. At the end-of-life of composite membranes, the recovery of the metals contained in the top layer could be attained through leaching processes, and therefore circumventing the need of pyrometallurgical processing. (ii) Leveraging the presence of the electric field produced between the emitter and the collector, the long-ranged orientation of the MOF could be controlled to produce particle alignment. Allahyarli *et al.*^53^ aligned various MOFs (*e.g.*, NU-1000, MIL-68(In) and MIL-53-NH_2_(Al)) using an electric field and liquid crystals as media; similarly, EHE could introduce this concept to produce membranes with aligned MOF particles.

### 3.5 Gas permeation

**Fig. 8:**
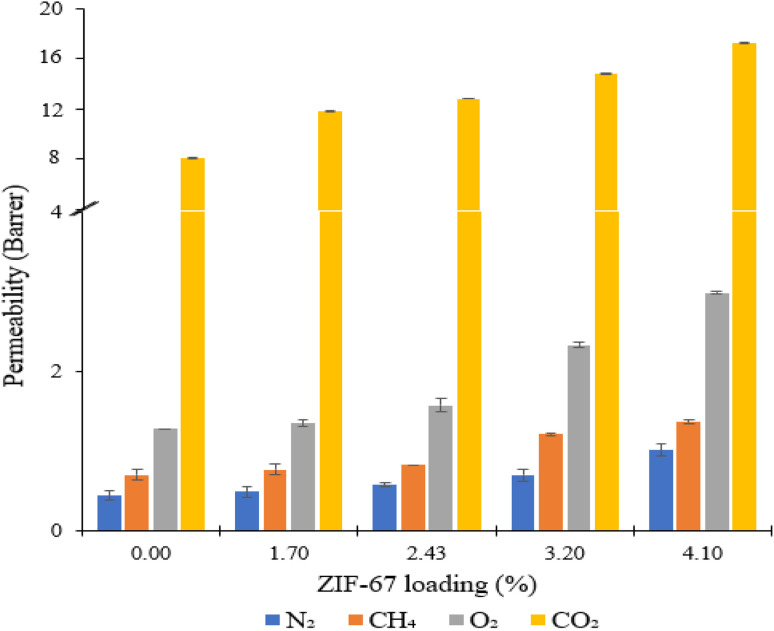
ZIF-67/CA asymmetric membrane gas permeability at different ZIF-67 loading.

**Fig. 9:**
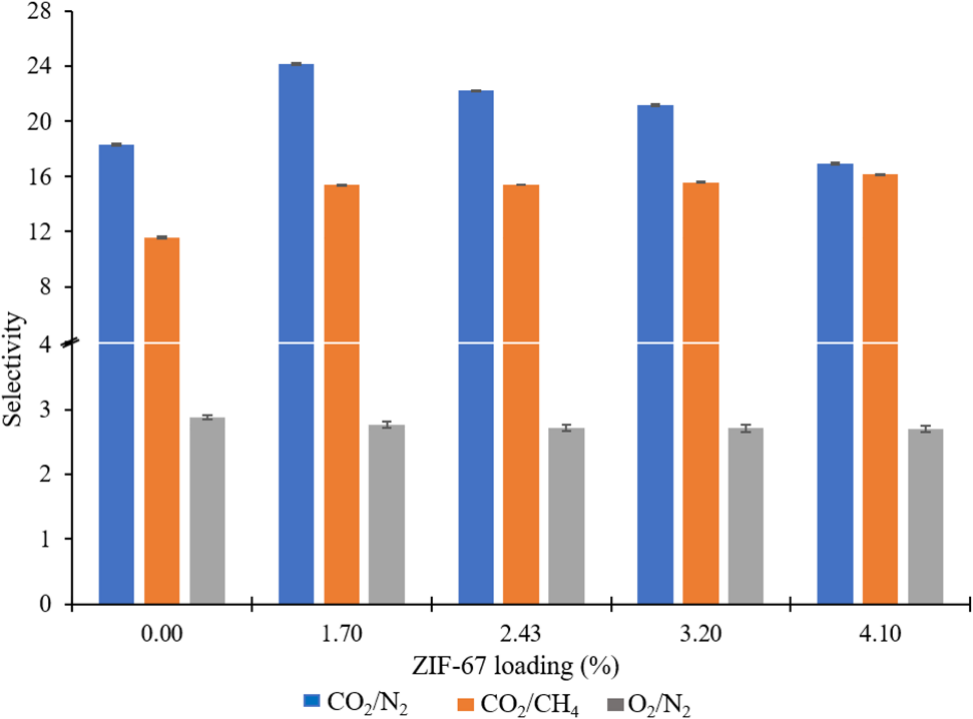
ZIF-67/CA asymmetric membrane gas selectivity at different ZIF-67 loading.

Gas permeation tests of ZIF-67/CA asymmetric membranes were performed for N_2_, O_2_, CH_4_ and CO_2_. The permeation analysis is performed at a feed pressure of 1 bar and temperature of 25 °C, employing the constant volume pressure increase approach. Fig. 8 shows the N_2_, O_2_, CH_4_ and CO_2_ permeances and the corresponding separation factors at a fixed temperature of 25 °C in Fig. 9. The performance of the ZIF-67/CA asymmetric membrane is influenced by ZIF-67 loading. The permeability of all gases (N_2_, O_2_, CH_4_ and CO_2_) increases as the ZIF-67 load increases. Compared to pristine CA membranes, the resultant permeability values were much higher when ZIF-67 was incorporated. At the highest load of 4.10 wt%, permeability values of 17.29 (±0.04), 2.75 (±0.01), 1.07 (±0.02) and 1.02 (±0.08) barrer were obtained for CO_2_, O_2_, CH_4_ and N_2_, respectively. Similarly, the CO_2_/CH_4_ ideal selectivity increased with the addition of ZIF-67, with a maximum value of 16 obtained at a loading of 4.10 wt%. The CO_2_/N_2_ ideal selectivity did not show any significant improvement with the addition of ZIF-67. While the O_2_/N_2_ ideal selectivity revealed that pristine CA membranes display a better performance than the asymmetric membranes. This observation is normal, considering the less affinity of the ZIF-67 to these gases.

Overall, the selectivity results obtained for CO_2_/CH_4_ and CO_2_/N_2_, shows that the asymmetric membrane outperformed the pristine CA membrane. This enhancement is attributed to the combined effect of the solution-diffusion and molecular sieving mechanisms exhibited by the asymmetric membrane. At the CA layer, like other dense polymeric membranes, the permeation of the gases through the membrane occurs *via* solution-diffusion.^54,55^ While the ZIF-67 layer has a molecular sieving capacity which is proven by the experimental gas transport results based on the Maxwell model reported in previous studies.^56^ More so, ZIF-67 has an affinity to CO_2_, and the kinetic diameter of ZIF-67 (0.34 nm) falls between that of CO_2_ (0.33 nm) and larger gases such as CH_4_ (0.38 nm) and N_2_ (0.364 nm). The above effects preferentially supported the permeability of CO_2_ which has a smaller molecular size compared to CH_4_ and N_2_ with larger molecular size.^25,26^ Likewise, CO_2_ has higher solubility compared to the other gases, hence, guaranteeing their high separation factor compared to those of O_2_/N_2_. This observation is consistent with those reported previously in the literature.^10,43^ The permeability and selectivity of the ZIF-67/CA asymmetric membrane was compared to those obtained from a ZIF-67/CA MMM at the same ZIF-67 loading (4.10 wt%). Table 1 shows that the permeability and selectivity of the asymmetric membrane is superior to those obtained from the MMM.

**Table 1:** Compared membrane gas permeability and selectivity^a^

ZIF-67 loading (wt%)		Permeability (barrer)				Selectivity		
		N_2_	CH_4_	O_2_	CO_2_	CO_2_/N_2_	CO_2_/CH_4_	O_2_/N_2_
A*	0.00	0.45 ± 0.07	0.71 ± 0.07	1.28 ± 0.00	8.16 ± 0.04	18.33	11.57	2.84
B*	4.10	1.02 ± 0.08	1.07 ± 0.02	2.75 ± 0.01	17.29 ± 0.04	16.95	16.16	2.70
C*	4.10	0.84 ± 0.05	1.39 ± 0.01	1.39 ± 0.02	11.14 ± 0.01	13.34	8.01	1.65

a A* = pure CA membrane; B* = ZIF-67/CA asymmetric membrane; C* = ZIF-67/CA mixed matrix membrane.

### 3.6 Molecular simulations and structural changes

**Fig. 10:**
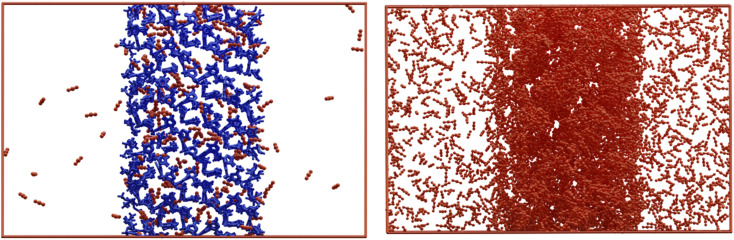
The simulation cell at a given frame of a ZIF-67 slab packed with 200 molecules of CO_2_ (left), and the combined trajectories of CO_2_ over all frames of a 2 ns simulation (right), where ZIF-67 has been removed for clarity.

To further understand the improved performance of the asymmetric membrane compared to the MMM, atomistic studies were performed on the ZIF-67 and CA systems, in addition to a composite system containing both components. Experimentally, the presence of ZIF-67, in either an asymmetric or MMM, increases the permeability of the membrane to all gases tested. This is most notable for CO_2_, where the permeability value is more than doubled in an asymmetric membrane compared to CA alone. This improvement may be attributed to the positive interaction of the ZIF-67 with the permeate, where gas molecules are preferentially drawn to the surface of the ZIF and retained in the porous structure. This behaviour is clear when visualising the combined trajectory of CO_2_; here, a higher concentration of CO_2_ is observed within the ZIF structure, compared to the layer of vacuum surrounding the zeolitic slab (Fig. 10). The increased absorption of permeate gases into the ZIF slab from vacuum is the opposite behaviour from what we have observed when analogous simulations are performed using a layer of polymer. In our previous studies,^57^ we have demonstrated that while attraction exists between gas molecules and polymer – leading to adherence to the polymer slab surface – no penetration is observed into the polymer layer throughout a MD simulation. The increased permeability values of ZIF-containing composite membranes can therefore be attributed to this increased absorption, which draws penetrating gas molecules through higher barrier, CA layer.

While this effect explains the improvement of ZIF-67 composite systems relative to CA membranes, it does not account for the improved permeability of asymmetric membranes when compared to MMM of the same ZIF loading. To study the interaction between polymer and ZIF, a composite system was considered, wherein a slab of ZIF-67 was in contact with amorphous CA. The morphology and dynamics of CA chains in the composite system was then compared to that in a neat CA system, to understand the behaviour occurring on the nanoscale which contributes to the improved performance of the asymmetric membrane (see S3 of the ESI†). The models suggest that presence of the ZIF-67 slab causes structural changes in the CA layer with less dynamic and mobile chains compared to their native arrangement without ZIF-67. Moreover, the polymer chains in immediate contact with the ZIF-67 surface are considerably denser than in the bulk system, with a 28% increase than the average CA bulk value. In fact, the overall variation in the axial density of CA in composite ZIF-67/CA membranes is 50% higher than in the neat system. This increase in density at the interface, combined with evidence that CA chains at the ZIF surface are elongated (in plane with the ZIF surface) and less mobile implies an increase in crystallinity. Finally, the trajectory of the MD simulations for the ZIF-67/CA system revealed that the ZIF-67 pores seem to attract the polymer chains into the imidazole window as shown in Fig. S8 (ESI).†

### 3.7 Mechanistic analysis of gas permeation in ZIF-67/CA asymmetric membranes

The atomistic insights may be used to rationalise the observed differences in barrier performance of the asymmetric membrane compared to the MMM. The use of EHE to deposit ZIF-67 ensures that a more consistent zeolitic layer is achieved, supported by CA, compared to a membrane achieved by mixing. As this ZIF layer lies normal to gas flux, a higher portion of incoming gas is forced to pass through the ZIF layer, where performance is boosted due to the favourable attraction between penetrant and the inorganic matrix. This phenomenon is therefore more pronounced in asymmetric membranes than in MMM, where a sporadic particle distribution means pathway for gas diffusion may bypass the ZIF altogether, and progress more slowly through the amorphous CA fraction.

**Fig. 11:**
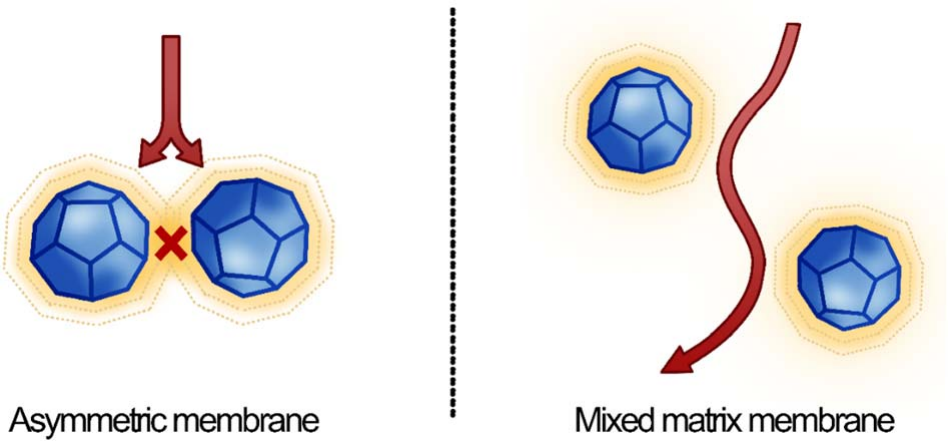
Illustration of the proposed mechanistic differences between gas diffusion pathway in asymmetric and MMM. Impermeable semicrystalline CA is depicted in yellow, and ZIF particles in blue. The diffusion pathway is shown as red arrows.

This study provides further possibilities to explain the enhancements of the asymmetric membrane. It is observed through simulation that the crystallinity of CA at the ZIF interface is increased. Densely packed elongated and immobilised chains in this partially crystalline layer provide fewer areas of free volume to host permeating gas molecules, which may cause oncoming gases to be pushed back into the amorphous bulk. In the case of MMM, as particles are more dispersed, this will manifest in fewer gas molecules passing through the zeolitic framework. In asymmetric membranes, as ZIF-67 is deposited normal to the gas flux, influx gas must pass through the concentrated ZIF layer, where spacing between ZIF particles is small. The increase in crystallinity of polymer which surrounds ZIF particles will effectively plug any defects which do exist between ZIF particles in the electrosprayed ZIF layer. This is because particles are closely packed, and therefore the option of bypassing the zeolitic framework *via* amorphous polymer is not possible in asymmetric membranes. In MMM, the larger spacing between dispersed ZIF particles ensures that gas molecules which have been rebuffed by the partially crystalline CA shell are more likely to proceed through surrounding amorphous polymer than through a neighbouring ZIF particle (Fig. 11). Although the alteration of CA polymer crystallinity at the ZIF-67/CA interface is demonstrated *via* simulations in this work; this phenomenon requires advanced characterization techniques for experimental observation, all of which are deemed outside the scope of this work.

## 4 Conclusions

In conclusion, a MOF-based asymmetric membrane for gas separation was successfully fabricated by applying the principle of EHE. ZIF-67 particles were electrosprayed to print a thin layer, while the CA polymer solution was cast on top of the ZIF-67 layer. The fabricated asymmetric membrane has a thickness of 20 μm and displayed a defect-free surface, even at high MOF loading. While the CO_2_/CH_4_ selectivity increased slightly with the addition of the ZIF-67 filler; the CO_2_/N_2_ selectivity recorded a maximum increase of 24%. The asymmetric membrane configuration resulted in improved CO_2_/N_2_, CO_2_/CH_4_, and O_2_/N_2_ selectivity. This enhancement was attributed to an improved interface interaction between the MOF and the CA polymer, good particle distribution engineered by electrospraying the MOF particles, and defect-free structure of the asymmetric membrane. In addition to the improved membrane separation, the EHE is outstanding compared to other methods of membrane fabrication, because it offers the opportunity for controlled dispersion and particle size distribution. It also enables the control of other functional properties that determine the overall membrane performance. Conceivably, the EHE techniques explored in this study for membrane fabrication can be leveraged in the manufacturing of composite membrane with improved separation performance. Using molecular simulations, we found that the presence of ZIF-67 particles generates structural changes to the CA polymeric chains at the interface – namely higher densities and reduced chain mobility (higher crystallinity). These features along with the adsorption of ZIF-67 can be leveraged in asymmetric configurations to yield composite membranes with an enhanced separation performance.

## Conflicts of interest

The authors declare no conflict of interest.

## Supplementary Material

RA-013-D3RA00995E-s001

## References

[cit1] Robeson M. L. (2008). The upper bound revisited. J. Membr. Sci..

[cit2] Carja I.-D., Tavares S. R., Shekhah O., Ozcan A., Semino R., Kale V. S., Eddaoudi M., Maurin G. (2021). Insights into the Enhancement of MOF/Polymer Adhesion in Mixed-Matrix Membranes via Polymer Functionalization. ACS Appl. Mater. Interfaces.

[cit3] He S., Zhu B., Jiang X., Han G., Li S., Lau C. H., Wu Y., Zhang Y., Shao L. (2021). Symbiosis-inspired de novo synthesis of ultrahigh MOF growth mixed matrix membranes for sustainable carbon capture. Proc. Natl. Acad. Sci. U. S. A..

[cit4] American S. , Scientific American, Scientific American Custom Media, 2018, available: https://www.scientificamerican.com/custom-media/pictet/a-material-to-save-the-world/, accessed 14 June 2022

[cit5] Beuscher U., Kappert E., Wijmans J. (2022). Membrane research beyond materials science. J. Membr. Sci..

[cit6] Chawla M., Saulat H., Khan M. M., Khan M. M., Rafiq S., Cheng L., Iqbal T., Rasheed M. I., Farooq M. Z., Saeed M., Ahmad N. M., Niazi M. B. K., Saqib S., Jamil F., Mukhtar A., Muhammad N. (2020). Gas membranes for CO_2_/CH_4_ and CO_2_/N_2_ gas separation. Chem. Eng. Technol..

[cit7] Li Y., He G., Wang S., Yu S., Pan F., Wu H., Jiang Z. (2013). Recent advances in the fabrication of advanced composite membranes. J. Mater. Chem. A.

[cit8] Venna S., Carreon M. (2015). Metal organic framework membranes for carbon dioxide separation. Chem. Eng. Sci..

[cit9] Chen W., Zhang Z., Hou L., Yang C., Shen H., Yang K., Wang Z. (2020). Metal-organic framework MOF-801/PIM-1 mixed-matrix membranes for enhanced CO_2_/N_2_ separation performance. Sep. Purif. Technol..

[cit10] Xu S., Zhou H., Jia H., Xu J. L. D., Zhang M., Qu Y., Ma W., Jiang P., Zhao W., Wang X. (2021). Preparation and high CO_2_/CH_4_ selectivity of ZSM-5/ethyl cellulose mixed matrix membranes. Mater. Res. Express.

[cit11] Suzuki T., Yamada Y. (2013). Synthesis and gas transport properties of novel hyperbranched polyimide–silica hybrid membranes. J. Appl. Polym. Sci..

[cit12] Tripathi B. P., Shahi V. K. (2011). Organic–inorganic nanocomposite polymer electrolyte membranes for fuel cell applications. Prog. Polym. Sci..

[cit13] Low Z. X., Chua Y. T., Ray B. M., Mattia D., Metcalfe I. S., Patterson D. A. (2017). Perspective on 3D printing of separation membranes and comparison to related unconventional fabrication techniques. J. Membr. Sci..

[cit14] Lipson H. and Kurman M., Fabricated: The New World of 3D Printing, John Wiley & Sons, 2013

[cit15] Chowdhury M. R., Steffes J., Huey B. D., McCutcheon J. R. (2018). 3D printed polyamide membranes for desalination. Science.

[cit16] Elsaidi S. K., Ostwal M., Zhu L., Sekizkardes A., Mohamed M. H., Gipple M., Hopkinson D. (2021). 3D printed MOF-based mixed matrix thin-film composite membranes. RSC Adv..

[cit17] Lahtinen E., Precker R. L., Lahtinen M., Hey-Hawkins E., Haukka M. (2019). Selective Laser Sintering of Metal-Organic Frameworks: Production of Highly Porous Filters by 3D Printing onto a Polymeric Matrix. ChemPlusChem.

[cit18] Basile A. and Favvas E. P., Current Trends and Future Developments on (Bio-) Membranes, Elsevier, 2018, pp. 625–643

[cit19] Baker R. W., Lokhandwala K. (2008). Natural Gas Processing with Membranes: An Overview. Ind. Eng. Chem. Res..

[cit20] Liu Y., Liu Z., Morisato A., Bhuwania N., Chinn D., Koros W. (2020). Natural gas sweetening using a cellulose triacetate hollow fiber membrane illustrating controlled plasticization benefits. J. Membr. Sci..

[cit21] Ahmad A., Jawad Z., Low S., Zein S. (2014). A cellulose acetate/multi-walled carbon nanotube mixed matrix membrane for CO_2_/N_2_ separation. J. Membr. Sci..

[cit22] Comito R. J., Fritzsching K. J., Sundell B. J., Schmidt-Rohr K., Dincă M. (2016). Single-Site Heterogeneous Catalysts for Olefin Polymerization Enabled by Cation Exchange in a Metal-Organic Framework. J. Am. Chem. Soc..

[cit23] Comito R. J., Wu Z., Zhang G., Lawrence J. A., Korzyński M. D., Kehl J. A., Miller J. T., Dincă M. (2018). Stabilized Vanadium Catalyst for Olefin Polymerization by Site Isolation in a Metal–Organic Framework. Angew. Chem., Int. Ed..

[cit24] Ahmadi M., Janakiram S., Dai Z., Ansaloni L., Deng L. (2018). Performance of Mixed Matrix Membranes Containing Porous Two-Dimensional (2D) and Three-Dimensional (3D) Fillers for CO_2_ Separation: A Review. Membranes.

[cit25] Matteucci S. , Yampolskii Y., Freeman B. and Pinnau I., Materials Science of Membranes for Gas and Vapor Separation, ed. Y. Yampolskii, B. D. Freeman and I. Pinnau, John Wiley & Sons, Ltd, 2006, pp. 1–47

[cit26] Rahul B., Anh P., Bo W., Carolyn K., Hiroyasu F., Michael O., Yaghi O. (2008). High-throughput synthesis of zeolitic imidazolate frameworks and application to CO_2_ capture. Science.

[cit27] Febriasari A., Suhartini M., Yunus A. L., Rahmawati R., Sudirman S., Hotimah B., Hermana R. F., Kartohardjono S., Fahira A., Permatasari I. P. (2021). Gamma Irradiation of Cellulose Acetate-Polyethylene Glycol 400 Composite Membrane and Its Performance Test for Gas Separation. Int. J. Technol..

[cit28] Omollo E., Zhang C., Mwasiagi J. I., Ncube S. (2016). Electrospinning cellulose acetate nanofibers and a study of their possible use in high-efficiency filtration. J. Ind. Text..

[cit29] Ahmad A., Jawad Z., Low S., Zein S. (2014). A cellulose acetate/multi-walled carbon nanotube mixed matrix membrane for CO_2_/N_2_ separation. J. Membr. Sci..

[cit30] Liu H., Hsieh Y.-L. (2002). Ultrafine Fibrous Cellulose Membranes from Electrospinning of Cellulose Acetate. J. Polym. Sci., Part B: Polym. Phys..

[cit31] Feng S., Bu M., Pang J., Fan W., Fan L., Zhao H., Yang G., Guo H., Kong G., Sun H., Kang Z., Sun D. (2020). Hydrothermal stable ZIF-67 nanosheets via morphology regulation strategy to construct mixed-matrix membrane for gas separation. J. Membr. Sci..

[cit32] Watson G. W., Kelsey E. T., de Leeuw N. H., Harris D. J., Parker S. C. (1996). Atomistic simulation of dislocations, surfaces and interfaces in MgO. J. Chem. Soc., Faraday Trans..

[cit33] Krokidas P., Castier M., Moncho S., Sredojevic D. N., Brothers E. N., Kwon H. T., Jeong H.-K., Lee J. S., Economou I. G. (2016). ZIF-67 Framework: A Promising New Candidate for Propylene/Propane Separation. Experimental Data and Molecular Simulations. J. Phys. Chem. C.

[cit34] Potoff J. J., Siepmann J. I. (2001). Vapor–liquid equilibria of mixtures containing alkanes, carbon dioxide, and nitrogen. AIChE J..

[cit35] Berendsen H. J. C., van-der Spoel D., van Drunen R. (1995). GROMACS: a message-passing parallel molecular dynamics implementation. Comput. Phys. Commun..

[cit36] Pronk S., Páll S., Schulz R., Larsson P., Bjelkmar P., Apostolov R., Shirts M. R., Smith J. C., Kasson P. M., van-der Spoel D., Hess B., Lindahl E. (2013). GROMACS 4.5: a high-throughput and highly parallel open-source molecular simulation toolkit. Bioinformatics.

[cit37] Abraham M. J., Murtola T., Schulz R., Páll S., Smith J. C., Hess B., Lindahl E. (2015). GROMACS: high performance molecular simulations through multi-level parallelism from laptops to supercomputers. SoftwareX.

[cit38] Purton J. A., Crabtree J. C., Parker S. C. (2013). DL_MONTE: a general purpose program for parallel Monte Carlo simulation. Mol. Simul..

[cit39] Qian J., Sun F., Qin L. (2012). Hydrothermal synthesis of zeolitic imidazolate framework-67 (ZIF-67) nanocrystals. Mater. Lett..

[cit40] Zhong G., Liu D., Zhang J. (2018). The application of ZIF-67 and its derivatives: adsorption, separation, electrochemistry and catalysts. J. Mater. Chem..

[cit41] Kamal H., Abd-Elrahim F., Lotfy S. (2014). Characterization and some properties of cellulose acetate-co-polyethylene oxide blends prepared by the use of gamma irradiation. J. Radiat. Res. Appl. Sci..

[cit42] Zhou Q., Zhang L., Zhang M., Wang B., Wang S. (2003). Miscibility, free volume behavior and properties of blends from cellulose acetate and castor oil-based. Polymer.

[cit43] Melgar V. M. A., Kwon H. T., Kim J. (2014). Direct spraying approach for synthesis of ZIF-7 membranes by electrospray deposition. J. Membr. Sci..

[cit44] Park K., Ni Z., Côté A., Choi J., Huang R., Uribe-Romo F., Chae H., O'Keeffe M., Yaghi O. (2006). Exceptional chemical and thermal stability of zeolitic imidazolate frameworks. Proc. Natl. Acad. Sci. U. S. A..

[cit45] Li Y., Liang F., Bux H., Feldhoff A., Yang W., Caro J. (2010). Molecular sieve membrane: supported metal-organic framework with high hydrogen selectivity. Angew. Chem..

[cit46] Yu C., Liang Y., Xue W., Zhang Z., Jia X., Huang H., Qiao Z., Mei D., Zhong C. (2021). Polymer-supported ultra-thin ZIF-67 membrane through in situ interface self-repair. J. Membr. Sci..

[cit47] Yin K., Zhang H., Yan Y. (2019). High efficiency of toluene adsorption over a novel ZIF-67 membrane coating on paper-like stainless steel fibers. J. Solid State Chem..

[cit48] Khan A., Ali M., Ilyas A., Naik P., Vankelecom I. F., Gilani M. A., Bilad M. R., Sajjad Z., Khan A. L. (2018). ZIF-67 filled PDMS mixed matrix membranes for recovery of ethanol via pervaporation. Sep. Purif. Technol..

[cit49] Tristantini D., Yunan A. (2018). Advanced characterization of microbeads replacement from cellulose acetate based on empty fruit bunches and dried jackfruit leaves. E3S Web Conf..

[cit50] Gopi S., Pius A., Kargl R. e. a. (2019). Fabrication of cellulose acetate/chitosan blend films as efficient adsorbent for anionic water pollutants. Polym. Bull..

[cit51] Sutrisna P. D., Savitri E., Gunawan M. A., Putri I. H. F., de-Rozari S. G. (2020). Synthesis, characterization, and gas separation performances of polysulfone and cellulose acetate-based mixed matrix membranes. Polym.-Plast. Technol. Mater..

[cit52] Castro-Munoz R., Agrawal K., Coronas J. (2020). Ultrathin permselective membranes: the latent way for efficient gas separation. RSC Adv..

[cit53] Allahyarli K., Reithofer M. R., Cheng F., Young A. J., Kiss E., Tan T. T. Y., Prado-Roller A., Chin J. M. (2022). Metal-organic framework superstructures with long-ranged orientational order via E-field assisted liquid crystal assembly. J. Colloid Interface Sci..

[cit54] Kaldis S. , Pantoleontos G. and Koutsonikolas D., Chapter 12 - Membrane Technology in IGCC Processes for Precombustion CO_2_ Capture, in Current Trends and Future Developments on (Bio-) Membranes, Elsevier, 2018, pp. 329–357

[cit55] Wijmans J. G., Baker R. W. (1995). The solution-diffusion model: a review. J. Membr. Sci..

[cit56] Fan H., Peng M., Strauss I. e. a. (2021). MOF-in-COF molecular sieving membrane for selective hydrogen separation. Nat. Commun..

[cit57] Lightfoot J., Buchard A., Castro-Dominguez B., Parker S. C. (2022). A comparative study of oxygen diffusion in PET and PEF using molecular modelling: computational insights into the mechanism for gas transport in bulk polymer systems. Macromolecules.

