# Supplementary material for: Asymmetric membranes for gas separation: interfacial insights and manufacturing
Source: RSC Adv. 2023 May 10;13(21):14198–209. doi: 10.1039/d3ra00995e (PMC10170239; doi:10.1039/d3ra00995e)
Supplement: RA-013-D3RA00995E-s001 [file RA-013-D3RA00995E-s001.pdf]

Electronic Supplementary Material (ESI) for RSC Advances.
This journal is © The Royal Society of Chemistry 2023

Asymmetric Membranes for Gas Separation: Interfacial Insights and Manufacturing

Sharifah Alkandari[a], Jasmine Lightfoot[a], Bernardo Castro-Dominguez[a*]

[a]Centre for Advanced Separations Engineering, Department of Chemical Engineering, University of Bath, Bath BA2 7AY, United Kingdom.

Supplementary Information

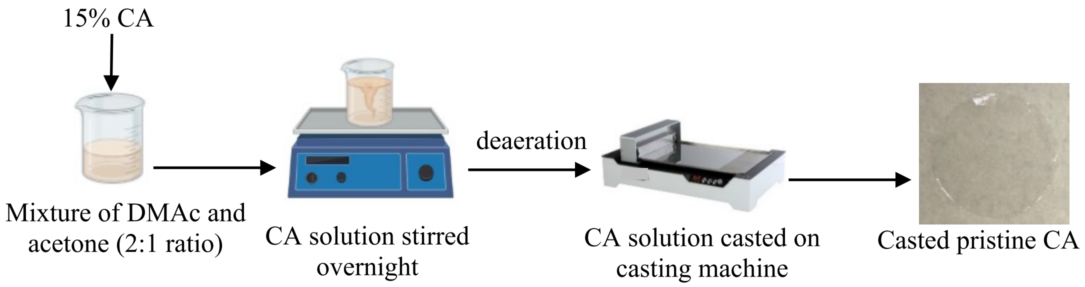

Figure S1 *– Schematic procedure for cellulose acetates solution preparation and casting.*

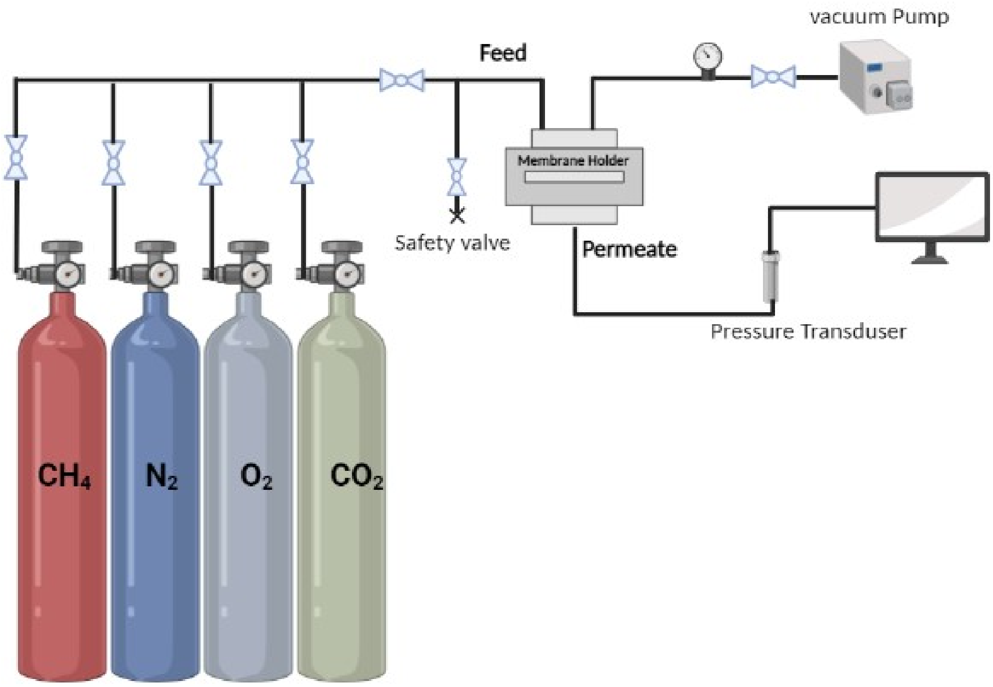

Figure S2 *– Setup of the gas permeation rig.*

* **Corresponding author:** Email: bcd28@bath.ac.uk; Office Number: +441225384946

S1. Gas Adsorption Properties of Membrane

Theoretically, gas adsorption in membranes occurs in steps. First, the gas molecules are transported from the bulk phase to the membrane surface. This is then followed by the diffusion of the gas into the surface of the membrane and finally, the gas solubilizes and subsequently gets adsorbed in the membrane matrix. However, the specific adsorption properties of these gases depend on the properties of the membrane material being studied.

The CA polymer used in this research is in the glassy polymer class, which normally follows the "dual-mode sorption model" of gas transport. This model is designed in two parts, the dense structure and molecular scale cavities, which shows a high tendency of CO_2 adsorption in pure cellulose acetate membrane material. This sorption isotherm of pure CA has been illustrated in several previous studies[1–3].

Furthermore, CO_2 is in general known to have a high affinity for certain types of materials, such as metal-organic frameworks (MOFs) including ZIF-67. This is because CO_2 has a small molecular size and a high quadrupole moment, which allows it to form strong interactions with the adsorption sites in these materials. O_2 and N_2 are both smaller than CO_2 and have a lower quadrupole moment, which makes them less likely to form strong interactions with adsorption sites. CH_4 is larger than all three of these gases and is therefore less likely to be adsorbed by materials with small pores[4–6]. The implication is that membrane consisting of MOF materials, the permeation of CO_2 will be higher compared to the other gases, and this was consistent with the observation in the gas permeation analysis in this study. Clearly the CO_2 permeation results from the respective membrane is validated by these well-established views about the materials used and their separation properties. Conceivably, the pure CA membrane is a nonporous material, as such may not show significant gas adsorption properties compared to the ZIF67/CA asymmetric membrane and ZIF67 / CA mixed matrix which have different gas adsorption properties due to the presence of the ZIF67 material.

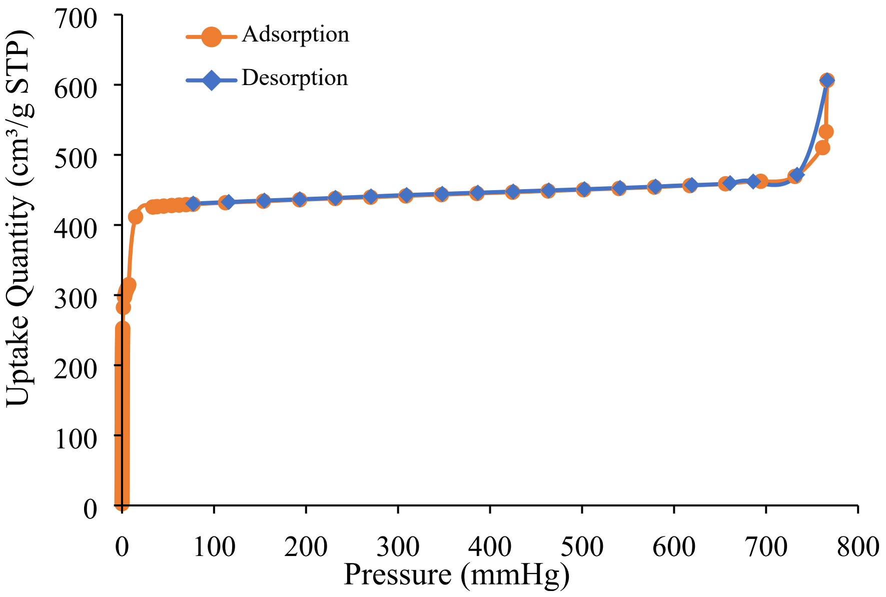

Figure S3 *– N_2 sorption isotherm of ZIF-67 collected at 77 K (BET=1,311 m^2/g)*

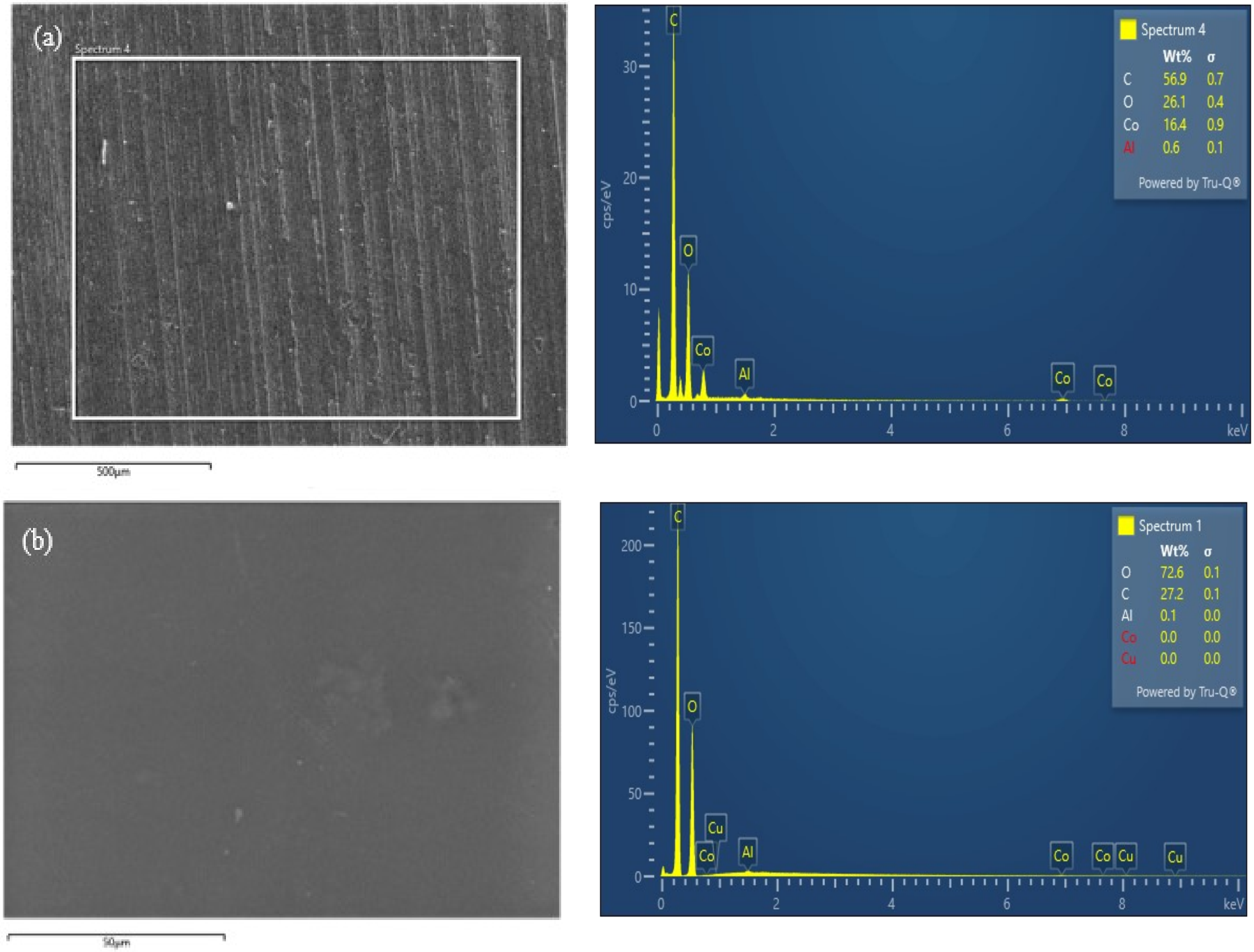

Figure S4 *– SEM-EDX analysis of the surface of ZIF-67/CA asymmetric membrane (a) top surface and (b) bottom surface.*

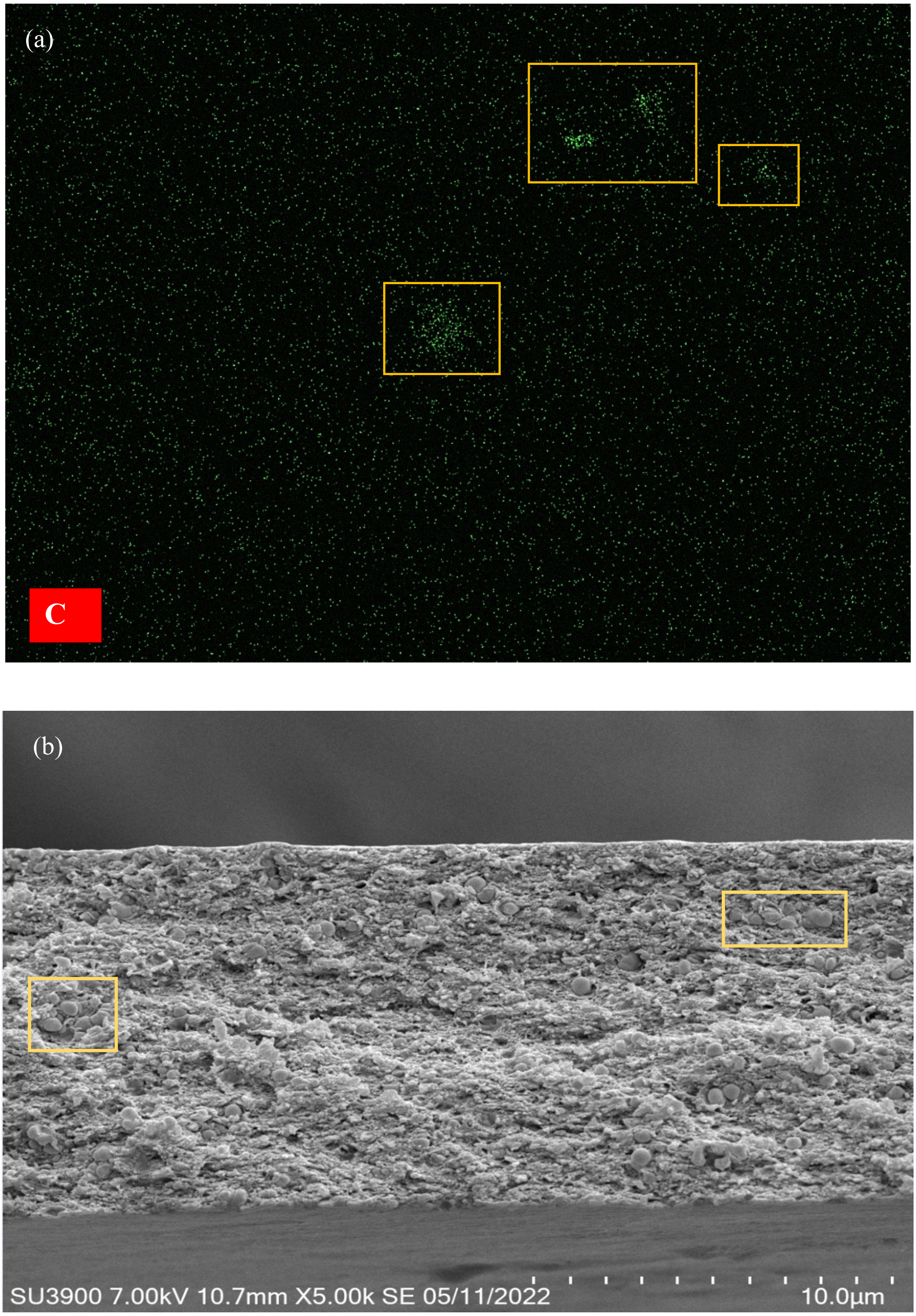

Figure S5 *– (a) EDX analysis of 4.10 wt.% ZIF-67/CA MMM surface and (b) SEM image cross section of 4.10 wt.% ZIF-67/CA MMM.*

S2. Simulations Methodology

Structures of ZIF-67 were generated from crystallographic data, derived from X-ray diffraction. Bulk and slab systems of the zeolite framework were built by growing the unit cell using METADISE[7]. Bulk systems were grown by a factor of 2 in the *x*, *y*, and *z* directions, such that each dimension of the simulation cell measured 33.8 A. respectively, with *α*, *β*, and *γ* angles of AA, BB, and GG reflecting those of the crystal unit cell. Slab systems of ZIF-67 were generated along the Miller Index (1 1 1), for the pores of the ZIF were normal to the generated surface, and the pore windows in-plane with the surface (Figure S6). Slab systems were monoclinic with values *a*, *b,* and *c* of 90 A, 71.7 A and 71.7 A respectively (with the slab surface normal to the *a* direction), and *α*, *β*, and *γ* angles of 60°, 90° and 90°. Recent studies to determine force field terms of ZIF-67 were used in this model,[8] and the TraPPE force field was implemented to model penetrating gas molecules.[9] An amorphous cellulose acetate system was built from 26 chains of 14 repeat units in length and modelled with the OPLS_AA force field.[10–11] This was equilibrated by first performing an energy minimisation on sparsely packed simulation cells, compressing the system at 750 K and 100 atm of pressure in the NPT ensemble, followed by 4 consecutive annealing cycles over 10 ns. Herein, a temperature ramp gradually alternates the simulation reference temperature between 298 K and 1200 K, before performing a final simulation in the NPT ensemble at room temperature and pressure, to allow for further system compression. In generating the composite ZIF-polymer system, 37 polymer chains were packed into a lengthened simulation cell containing a ZIF-67 slab. Equilibration of polymer in a composite system was achieved following the same procedure as in neat cellulose acetate systems, other than anisotropic pressure coupling was implemented in NPT ensembles. This ensured that the *b,* and *c* cell lengths and *α*, *β*, and *γ* angles of the ZIF crystal cell were maintained, while allowing for volume fluctuations in the *a* direction – being the axis orthogonal to the ZIF slab surface (see Figure S6) - to allow for compression of the polymer fraction. . The open-source molecular dynamics package, GROMACS[12–15] was used to perform molecular dynamics simulations. All equilibration simulations employed a Berendsen thermostat and barostat for temperature and pressure coupling.

Systems were validated by comparing the structural output of molecular simulation to experiment. Densities of cellulose acetate and ZIF-67, in both neat and composite systems, were quantified over 1 ns. Free volume calculations were performed by extracting the configurational output of molecular dynamics simulations and using the SCAN function of DL_MONTE,[16] to perform systematic grand canonical Monte Carlo insertions of a hydrogen probe molecule at regular intervals throughout the simulation cell. Coordinates where insertions were energetically favourable could be extracted, for visualisation of free volume in conjunction with the molecular structure which surrounds it. The calculation of density, dihedral distribution, free volume, radius of gyration and radial distribution function were performed in the NVT ensemble, employing the canonical Nosé-Hoover thermostat for temperature coupling. Radii of gyration of cellulose acetate molecules were computed from the GROMACS 'gyrate' function, where atoms are explicitly mass weighted. This is calculated by the following equation, where m_i is the mass of the atom *I*, and $\mathbf{r}_i$ is the position of the atom *i* with respect to the molecule's centre of mass:

$$Rg = \sqrt{\frac{\sum_i \|r_i\|^2 m_i}{\sum_i m_i}}$$

Axial radii components are given as mass-weighted root-mean-square values about the given axis, as a function of the radii components orthogonal to this axis. An example calculation is given in the following equation:

$$Rg_x = \sqrt{\frac{\sum_i \left(r_{i,y}^2 + r_{i,z}^2\right)^2 m_i}{\sum_i m_i}}$$

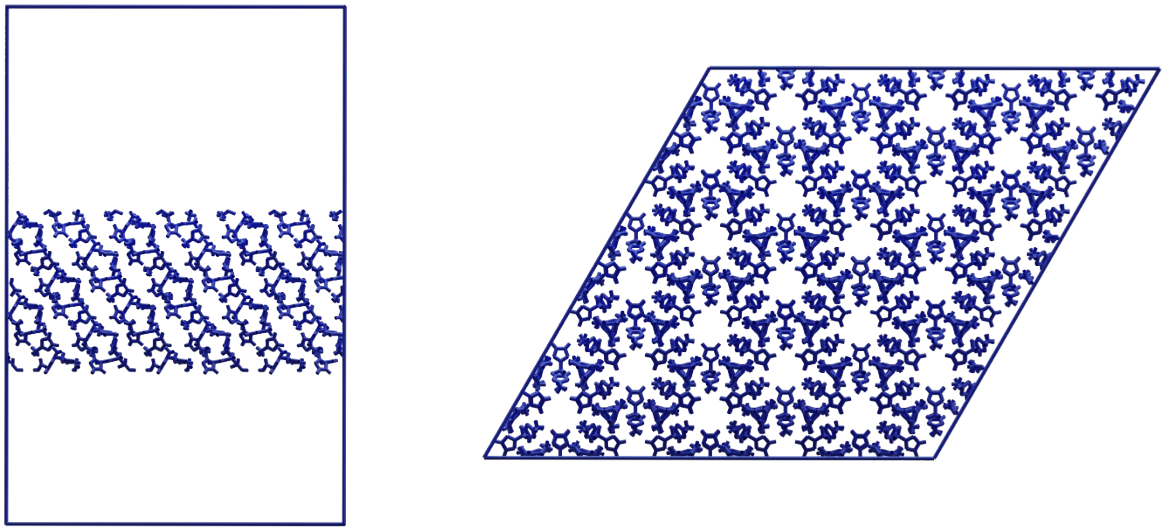

Figure S6 *– Slab of ZIF-67, generated along the (1 1 1) index such that the pores are aligned with the x axis.*

S3. Structural Analysis of Models

In the first instance, the structure of the ZIF-67 crystal unit cell was analysed, and from which all subsequent zeolitic systems were generated. The morphology of the central spherical pore was observable through free volume calculations, achieved by subjecting the unit cell to grand canonical Monte Carlo simulation. By analysing coordinates where hydrogen insertion is favourable, the cage like structure of ZIF-67 can be seen. The central spherical pore is accessible through eight narrow channels, which run through the aperture made by the imidazole heterocyclic moieties arranged in a solalite-like framework (**Figure S7**). The pore volume was found to have a diameter of 11.63 A, exactly matching that reported by Tsao and coworkers.[17] A calculated window aperture (*wa*) of 6.8 A, and a window height (*h*) of 7.95 A was also found to be consistent previous studies (**Figure S8**).

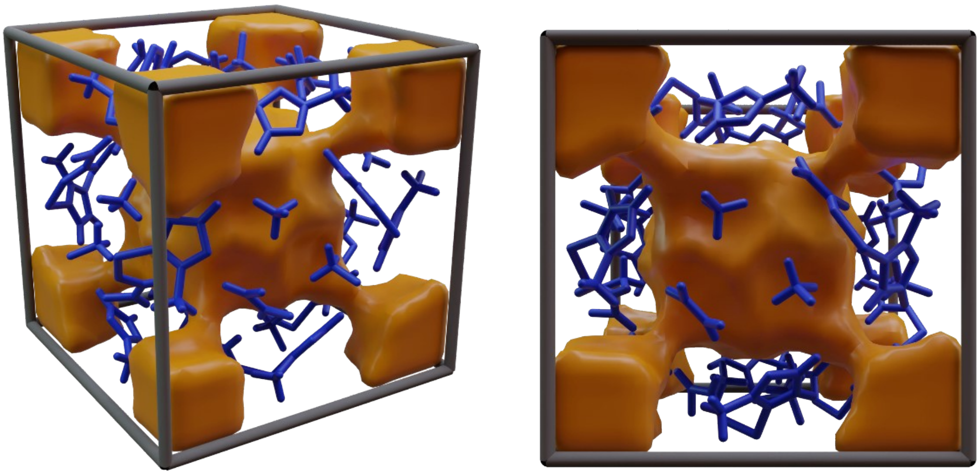

Figure S7 *– Free volume within a ZIF-67 unit cell, where a central spherical pore volume is accessible via eight narrow channels. Free volume was calculated from grand canonical Monte Carlo simulation.*

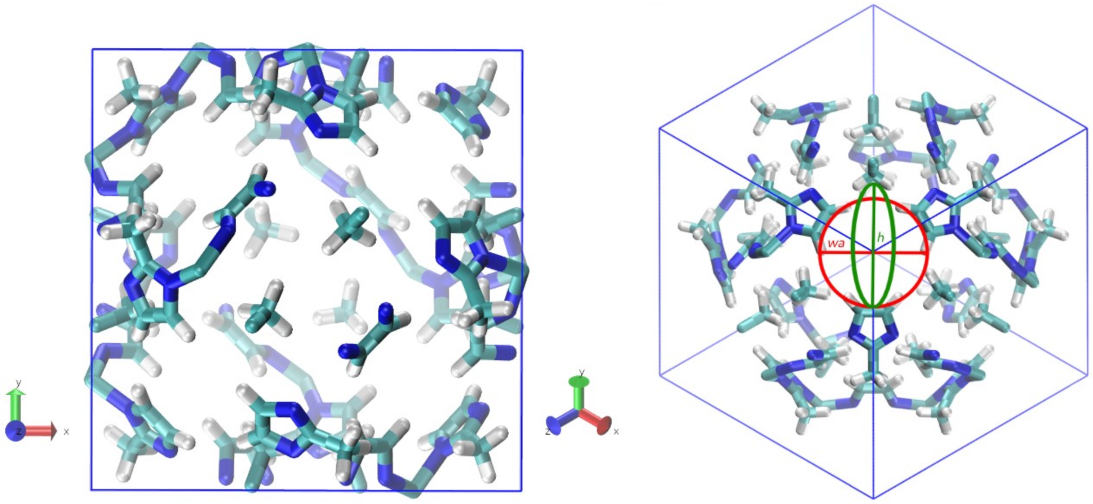

Figure S8 – *Window aperture (wa) and window height (h) calculated from the crystal structure of ZIF-67.*

S4. Structural Changes in Neat and Asymmetric Membranes

The radius of gyration was analysed for cellulose acetate chains in neat and composite systems. It was observed that the radius of gyration was approximately 0.5 nm greater for cellulose acetate in the ZIF-containing system when compared to in the neat polymer. The gyration radius was also seen to fluctuate considerably less in the composite system, with a standard deviation across the simulation approximately half the value of that in the neat system (0.017 and 0.028 nm respectively). These results indicate that the presence of the ZIF-67 slab causes structural changes in the cellulose acetate layer; whilst the larger radius of gyration demonstrates that each chain is more elongated, the lower variation indicates that these are less dynamic and mobile compared to their native arrangement without ZIF. Indeed, by analysing the radii components orthogonal to each axis, the direction of alignment of cellulose acetate chains in ZIF-composite systems is in plane with the ZIF surface, bringing about the largest radius of gyration about the x-axis. The radius of gyration in the x-axis is significantly greater than in the y- or z- directions, at 3.052 nm, 2.300 nm and 2.563 nm respectively. It is also in this direction where chains experience the most subdued movement, as evidenced by the lowest levels of variation throughout the simulation. As expected, the radius of gyration in neat systems show no significant difference in any direction, due to the homogeneity of the system. The alignment of the chains normal to the x-axis is therefore attributed to an attraction between ZIF surface and polymer, which causes polymer chains to spread out to maximise the positive interaction. These results are presented in Table S1 and Figure S9.

Table S1 **-** Radius of gyration of cellulose acetate chains in a neat polymer, and polymer-ZIF composite system.

	Polymer system	Composite system
Rg / nm	**2.881**	**3.253**
Deviation in Rg / nm	**0.028**	**0.017**
Rg(x) / nm	2.364	**3.052**
Deviation in Rg(x) / nm	0.034	**0.010**
Rg(y) / nm	2.285	2.300
Deviation in Rg(y) / nm	0.018	0.022
Rg(z) / nm	2.408	2.563
Deviation in Rg(z) / nm	0.030	0.022

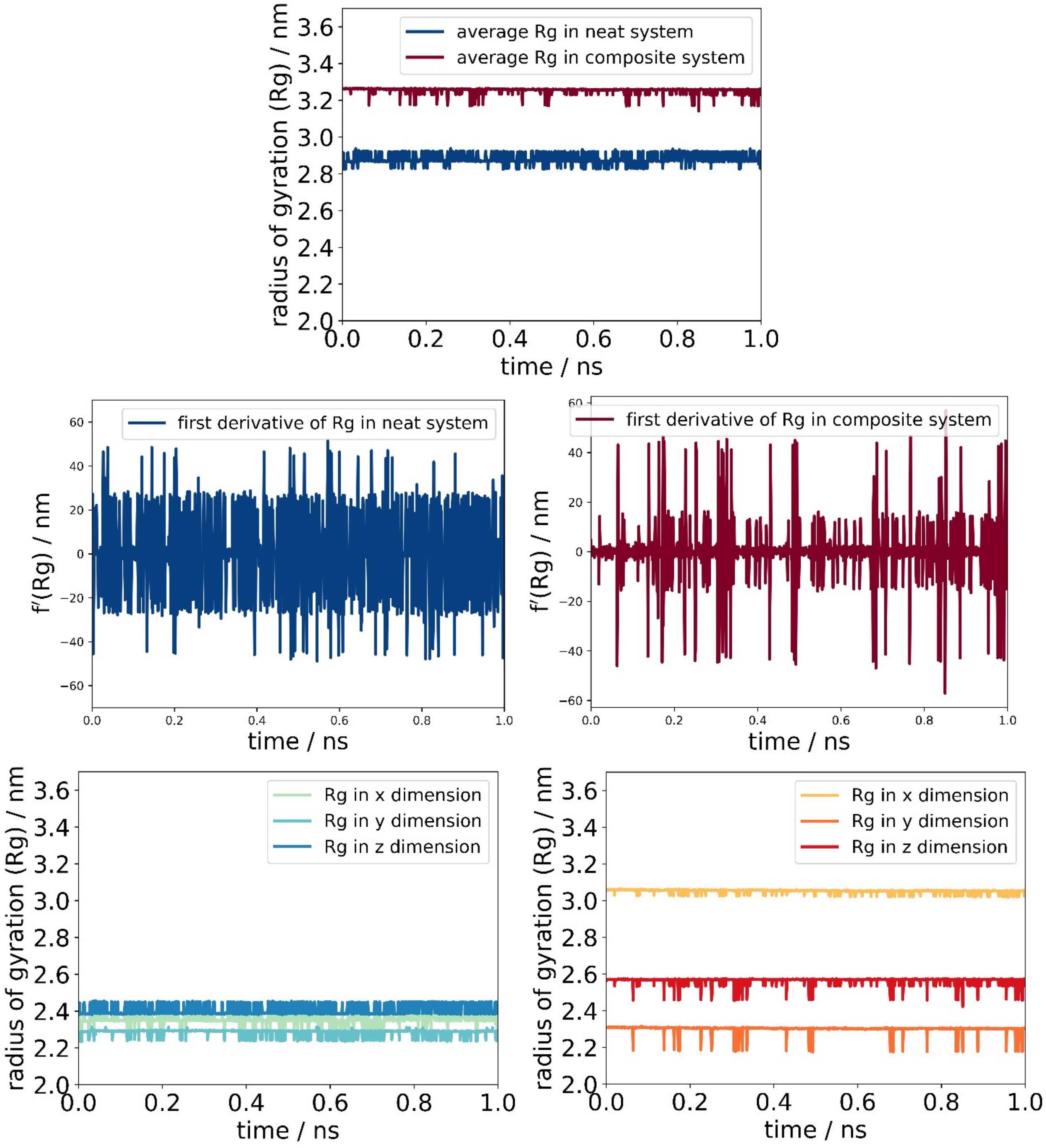

Figure S9 – Average radius of gyration (Rg) of cellulose acetate chains in neat polymer and polymer composite systems (top centre), derivative of Rg in neat polymer (middle left) in polymer composite (middle right), and the axial radii of gyration about the *x*, *y*, and *z* axes in neat polymer (bottom left) and polymer composite (bottom right) systems.

The density of the polymer layer was also quantified and compared to that of the neat system and experiment. In a neat system, the average density was calculated as 1.23 g.cm^{-3}, demonstrating good agreement with the experimental value of 1.28 g.cm^{-3} for an amorphous sample.[14] In a neat cellulose acetate system, density is seen to fluctuate around this value, with a standard deviation of 0.10 g.cm^{-3} / 8 % depending on the average density along the *x*-axis. As the system is homogeneous, this relatively low variation and lack of pattern by location is expected. When the same density analysis is performed on the cellulose acetate layer of the

composite systems, along the *x*-axis which runs orthogonal to the ZIF surface, greater structural variations are observed (Figure S10). Polymer chains in immediate contact with the ZIF surface are considerably more dense than in the bulk system, attaining a maximum density of 1.58 g.cm^{-3}, 0.35 g.cm^{-3} / ~28 % higher than the average bulk value. Overall, variation in the axial density of cellulose acetate in composite is 50 % higher than in the neat system, with a calculated deviation of 0.15 g.cm^{-3}. This higher degree of variation is attributed to the dense polymer coordination shell which is seen to form on the ZIF surface. This phenomenon has been previously remarked in our previous studies into the interactions of PLA chains on nanoclay surfaces. Indeed, a visual inspection of molecular dynamics trajectories shows evidence of neighbouring elongated cellulose acetate chains bunding and imparting crystallinity to the polymer phase at the interface.

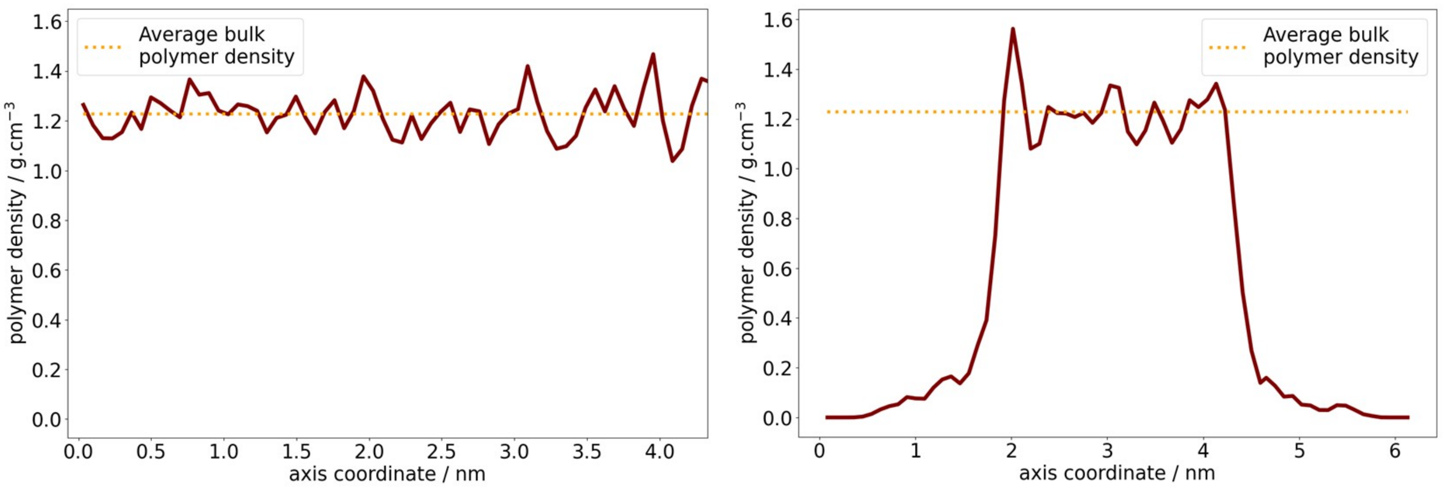

Figure S10 – Density fluctuations of cellulose acetate in a neat amorphous system (left) and in a composite system in contact with ZIF-67.

S5. Cellulose Acetate and ZIF-67 Interphase

Visual inspection of the trajectory of the MD simulation for the cellulose acetate/ZIF-67 system reveals interesting insights into the interaction of chains with ZIF pore windows. In the same manner than ZIF-67 pores are seen to attract and draw permeating carbon dioxide molecules into its pores, polymer chains are also seen to interact with the imidazole window. During system equilibration, this results in cellulose acetate chain ends entering surficial ZIF pores (Figure S11). This behaviour is likely to be exaggerated in an atomistic study, as there is a higher ratio of chain ends per molecule due to limitations in simulating experimental molecular weights of polymers. Figure S12 shows the free volume distribution of the ZIF and the CA membranes.

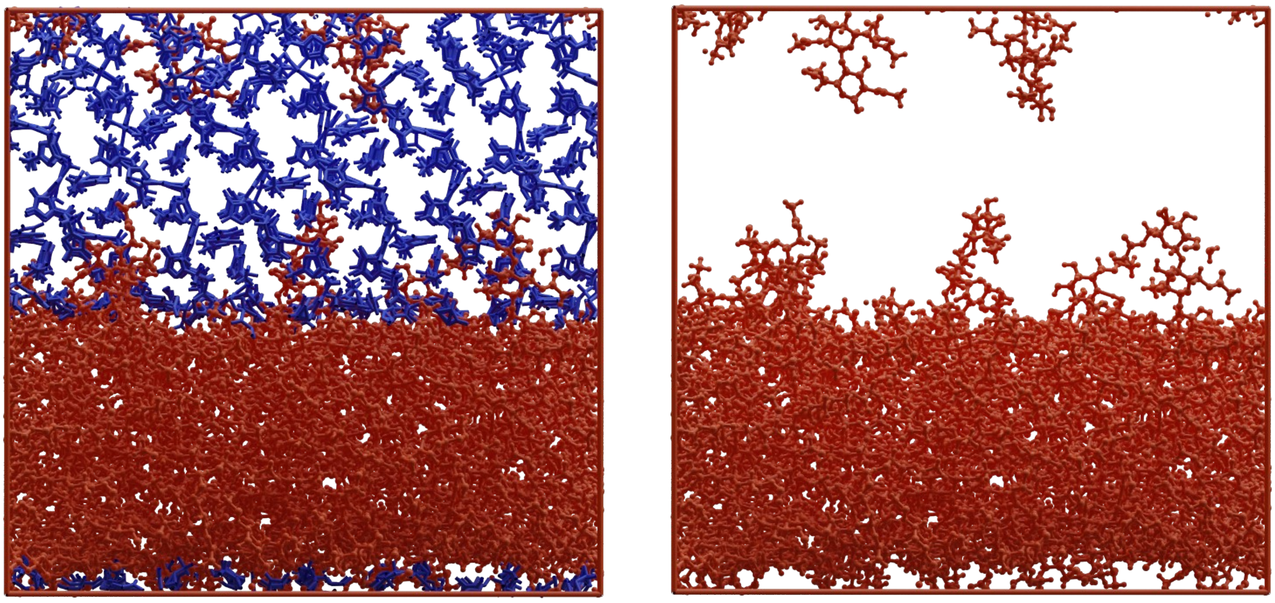

Figure S11– *ZIF-67/ cellulose acetate composite system (left) where ZIF has been removed for visibility (right), showing the interaction of polymer chains with ZIF pores.*

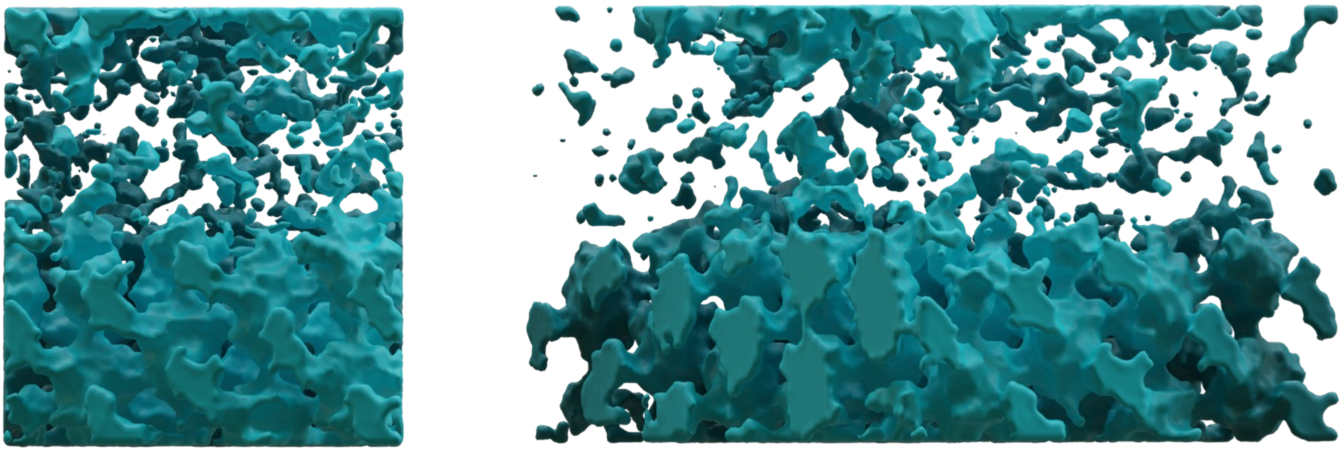

Figure S12 – *Free volume distribution in ZIF/CA system.*

References:

1. Hafeez, S, Fan, X & Hussain, A., (2015). 'A Kinetic Study of CO2 Adsorption in Cellulose Acetate Membranes', International Journal of Environmental Science and Development, vol. 6, no. 10, pp. 755-759. https://doi.org/10.7763/IJESD.2015.V6.694
2. Stern, S.A. and De Meringo, A.H. (1978), Solubility of carbon dioxide in cellulose acetate at elevated pressures. J. Polym. Sci. Polym. Phys. Ed., 16: 735-751. https://doi.org/10.1002/pol.1978.180160415
3. Sada, E., Kumazawa, H., Yoshio, Y., Wang, S.-T. and Xu, P. (1988), Permeation of carbon dioxide through homogeneous dense and asymmetric cellulose acetate membranes. J. Polym. Sci. B Polym. Phys., 26: 1035-1048. https://doi.org/10.1002/polb.1988.090260508
4. Ethiraj J., Palla S., Reinsch H., (2020). Insights into high pressure gas adsorption properties of ZIF-67: Experimental and theoretical studies. Microporous and Mesoporous Materials 294 (2020) 109867. https://doi.org/10.1016/j.micromeso.2019.109867
5. Feng S., M. Bu, J. Pang, W. Fan, L. Fan, H. Zhao, G. Yang, H. Guo, G. Kong, H. Sun, Z. Kang and D. Sun, (2020). “Hydrothermal stable ZIF-67 nanosheets via morphology regulation strategy to construct mixed-matrix membrane for gas separation,” Journal of membrane science, 593, 117404. https://doi.org/10.1016/j.memsci.2019.117404
6. H. Yang, X.W. He, F. Wang, Y. Kang, J. Zhang, (2012). Doping copper into ZIF-67 for enhancing gas uptake capacity and visible-light-driven photocatalytic degradation of organic dye, J. Mater. Chem. 22, 21849–21851.
7. G. W. Watson, E. T. Kelsey, N. H. de Leeuw, D. J. Harris and S. C. Parker, J. Chem. Soc. Faraday Trans., 1996, 92(3), 433–438, https://doi.org/10.1039/FT9969200433
8. P. Krokidas, M. Castier, S. Moncho, D. N. Sredojevic, E. N. Brothers, H. T. Kwon, H.-K. Jeong, J. S. Lee and I. G. Economou, J. Phys. Chem. C, 2016, 120, 8116–8124, https://doi.org/10.1021/acs.jpcc.6b00305
9. J. J. Potoff and J. I. Siepmann, AIChE J., 2001, 47, 1676–1682. https://doi.org/10.1002/aic.690470719
10. W. Damm, A. Frontera, J. Tirado–Rives and W. L. Jorgensen, J. Comput. Chem., 1997, 18, 1955–1970, https://doi.org/10.1002/(SICI)1096-987X(199712)18:16<1955::AID-JCC1>3.0.CO;2-L
11. W. L. Jorgensen, D. S. Maxwell and J. Tirado-Rives, J. Am. Chem. Soc., 1996, 118, 11225–11236, https://doi.org/10.1021/ja9621760
12. H. J. C. Berendsen, D. van der Spoel and R. van Drunen, Comput. Phys. Commun., 1995, 91, 43–56, https://doi.org/10.1016/0010-4655(95)00042-E
13. S. Pronk, S. Páll, R. Schulz, P. Larsson, P. Bjelkmar, R. Apostolov, M. R. Shirts, J. C. Smith, P. M. Kasson, D. van der Spoel, B. Hess and E. Lindahl, Bioinformatics, 2013, 29, 845–854, https://doi.org/10.1093/bioinformatics/btt055.
14. M. J. Abraham, T. Murtola, R. Schulz, S. Páll, J. C. Smith, B. Hess and E. Lindahl, SoftwareX, 2015, 1–2, 19–25, https://doi.org/10.1016/j.softx.2015.06.001
15. J. A. Purton, J. C. Crabtree and S. C. Parker, Mol. Simul., 2013, 39, 1240–1252, https://doi.org/10.1080/08927022.2013.839871
16. C.-Y. Wang, C.-M. Chou, P.-S. Tseng and C.-S. Tsao, J. Chinese Chem. Soc., 2021, 68, 500–506, https://doi.org/10.1002/jccs.202000561
17. D. M. Polyukhov, A. S. Poryvaev, A. S. Sukhikh, S. A. Gromilov and M. V Fedin, ACS Appl. Mater. Interfaces, 2021, 13, 40830–40836, https://doi.org/10.1021/acsami.1c12166
